# Anisotropic Microstructure and Chemo-Optical Properties of Fluorocarbon Films Deposited by Ablative Pulsed Plasma Thruster Plumes

**DOI:** 10.3390/polym18202562

**Published:** 2026-10-17

**Authors:** Rui Zhang, Zhen He, Yingjie Zhao, Wenxiong Xi

**Affiliations:** 1 School of Engineering Science, Shandong Xiehe University, Jinan 250107, China; hezhen@sdxiehe.edu.cn (Z.H.); zhaoyingjie@sdxiehe.edu.cn (Y.Z.); 2 Research Institute of Aerospace Technology, Central South University, Changsha 410012, China; 13739076081@163.com

**Keywords:** ablative pulsed plasma thruster, fluorocarbon film, plasma dynamics, plasma–surface interaction, chemo-optical properties, plume contamination

## Abstract

Ablative pulsed plasma thruster (APPT)-induced fluorocarbon contamination severely degrades microsatellite optics and solar panels, but the spatial heterogeneity of its chemo-optical properties and underlying plasma-film mechanisms remain poorly understood. In this work, X-ray photoelectron spectroscopy (XPS), scanning probe microscopy (SPM), scanning electron microscopy (SEM), and ultraviolet-visible (UV-Vis) spectrophotometry were used to characterize the chemical structure, morphology, thickness, and chemo-optical properties of PTFE-derived fluorocarbon films, while high-speed imaging and triple Langmuir probe diagnostics were used to map plume conditions governing film formation. The film *F/C* ratio (0.6–1.0) is far lower than that of pristine PTFE (2.0), controlled by competition between energetic ion sputtering and delayed neutral C_2_F_4_ deposition. In the electrode-perpendicular plane, the *F/C* ratio reaches a minimum ~0.6 at 30°, corresponding to a transmittance of <20% at 550 nm; anode-side films exhibit lower *F/C* ratios and transmittance yet higher reflectance than cathode-side counterparts. Films in the electrode-parallel plane show quasi-symmetric bond distributions and higher *F/C* ratios and transmittance, with *F/C* minima at ±15°. By correlating plasma anisotropy (cathode-biased electron density and anode-biased electron temperature) with film chemistry and optics, we identify this ion–neutral competition as the origin of angle-dependent film properties. This work provides design guidelines: optics within 30° of the thruster axis in the perpendicular plane require active protection, enabling quantitative anisotropic contamination risk assessment for microsatellites.

## 1. Introduction

**Figure 1:**
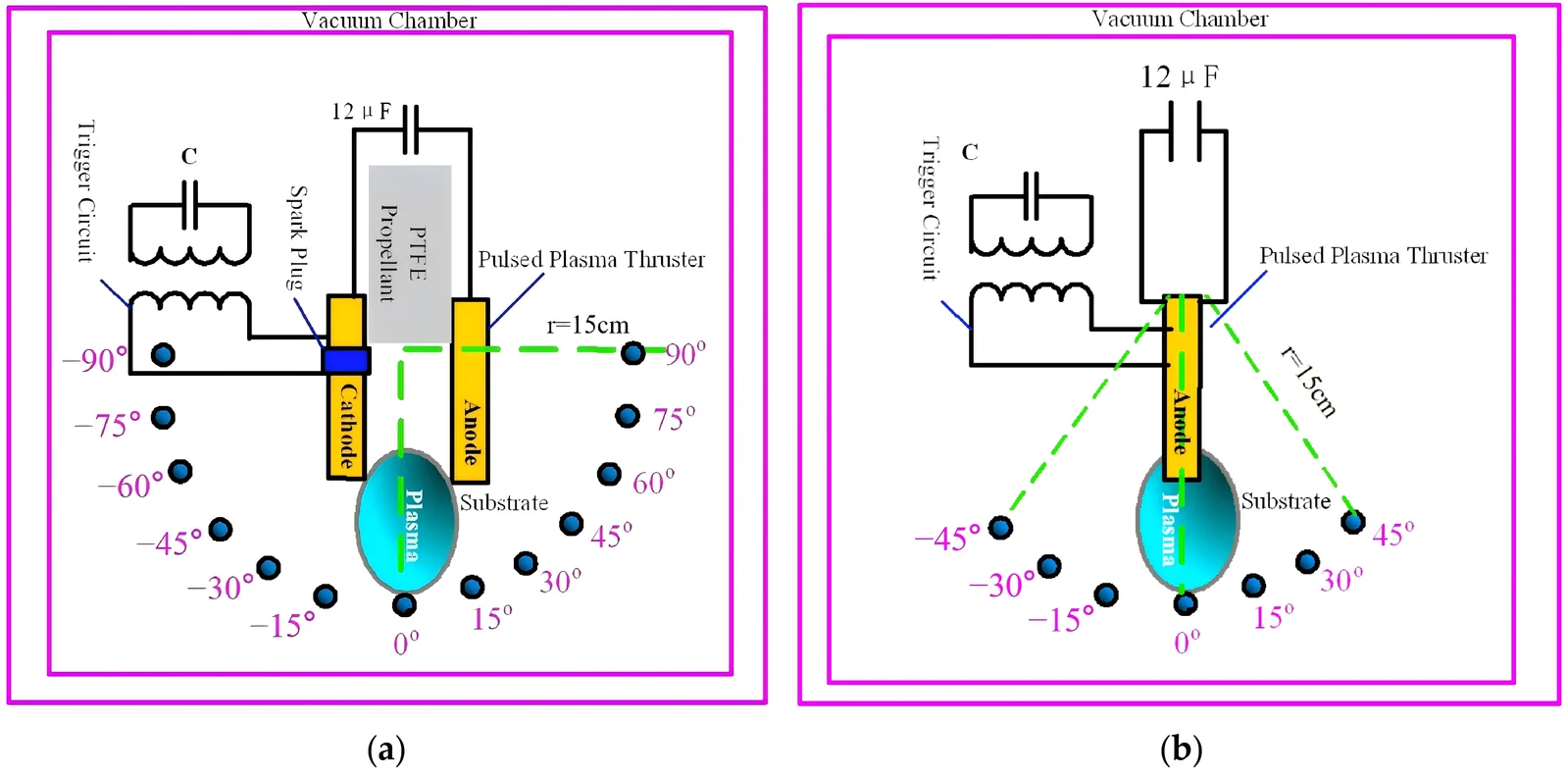
Substrate placement for film deposition. (**a**) Electrode-perpendicular plane. (**b**) Electrode-parallel plane.

Ablative pulsed plasma thrusters (APPTs) are core electric propulsion devices for microsatellite attitude control, the task of maintaining or adjusting a satellite’s orientation in space. They are favored for this application due to their simple structure and high specific impulse [1,2]. In an APPT, a spark plug triggers a high-voltage discharge across the surface of the solid polytetrafluoroethylene (PTFE) propellant. The discharge current produces thermal flux that pyrolyzes the PTFE propellant at the surface, and the resulting ablation products are dissociated and ionized to form a current-carrying, partially ionized plasma sheet. This plasma sheet is then accelerated by the combined effect of electromagnetic and aerodynamic forces, generating a high-velocity exhaust plume composed of plasma and neutral species (Figure 1). Although this process yields the required thrust, it inevitably gives rise to plume-borne contamination: fluorocarbon ejecta deposit onto spacecraft surfaces, degrading solar panel efficiency, altering the transmittance and reflectance of optical sensors, and causing imaging distortion [3,4,5]. Addressing this contamination requires a fundamental understanding of how spatially varying plume dynamics control film formation and final properties—a critical knowledge gap.

Characterizing APPT contamination requires establishing a direct link between plasma plume dynamics and film formation behavior [6,7]. Langmuir probes enable direct quantification of key plasma parameters, i.e., electron number density (*n_e_*) and temperature (*T_e_*), which reflect changes in plasma characteristics. Previous studies have reported that *T_e_* decreases with distance from the APPT outlet [8,9,10], and *n_e_* exhibits a cathode-biased distribution [11].

Beyond quantifying the fundamental plasma parameters, identifying the specific species present in the plume is equally critical for understanding deposition chemistry. Optical emission spectroscopy (OES) has been widely used to identify plasma species in APPT plumes. For instance, Markusic et al. [12] detected carbon ions (C^+^), fluorine ions (F^+^), and C_2_ neutrals from a laboratory-model pulsed plasma thruster (XPPT-1). Koizumi et al. [13] further employed Swan-band spectra (emission bands of C_2_ molecules used for diagnosing neutral species) to achieve spatial resolution of emissions and distinguish ions from neutrals. Furthermore, Wu et al. [14,15] employed time-resolved OES to track plasma recombination processes, whereas Schönherr et al. [16] systematically revealed the asymmetric plasma distribution characteristics between APPT electrodes. Nevertheless, a spatially resolved correlation between these plasma parameters and the resulting film composition, bonding, and optical properties has never been established. This missing link hampers predictive contamination modeling.

Concerning thin-film characterization, research on APPT deposited contaminated films has made some progress over the past few decades, whereas the fundamental understanding of the film formation-property correlation remains insufficient. Early characterizations based on Fourier Transform Infrared Spectroscopy (FTIR) were severely restricted by inherent limitations in spectral resolution [17]. Similarly, some preliminary XPS research (Rudolph and Jones [18]) incorrectly attributed fluorine-rich deposited films to PTFE-like structural components owing to instrumental performance limitations. Thereafter, Arrington et al. [19] confirmed the fluorocarbon nature of APPT deposits via Auger Electron Spectroscopy (AES) but lacked angular resolution and plasma-film correlation. Accordingly, the microstructure–property relationships governing optical performance have been largely overlooked.

Broader research on fluorocarbon and diamond-like carbon (DLC) films can provide valuable guidance. Kimura et al. [20] used an APPT to deposit fluorinated DLC films, with a focus on how discharge energy influences film hardness and structure—a research topic that differs from our focus on contamination. More importantly, studies on functional fluorocarbon films [21,22,23,24] have firmly established a core paradigm: plasma deposition parameters regulate chemical bonding (e.g., *F/C* ratio) and nanostructure, which then ultimately determine the optical transmittance and reflectance of the films. This paradigm offers a solid theoretical foundation for our investigation into unintentional contamination from APPT plumes.

Based on the above comprehensive analysis, we identify three interconnected gaps in APPT contamination research:

(a) Spatial heterogeneity: No systematic mapping of film composition, nanostructure, and chemo-optical properties exists across distinct plume angles, especially comparing the electrode-perpendicular and electrode-parallel planes.
(b) Plasma-film correlation: The pathways through which *n_e_*, *T_e_*, and neutral fluxes govern *F/C* and bond configuration (C-C, CF_2_, etc.) remain unclear.
(c) Chemo-optical anisotropy: The angular dependence of transmittance and reflectance—critical for assessing spacecraft optical degradation—has not been quantitatively linked to plasma conditions.

To bridge these gaps, this work recognizes that APPT-induced spacecraft contamination originates from the deposition of PTFE-derived fluorocarbon films. Plasma diagnostics are applied to elucidate film-formation mechanisms, while complementary material-characterization techniques probe the chemical structure, morphology, and resulting optical properties of these polymer-like films. Specifically, we adopt the plasma–structure–property paradigm with a suite of complementary diagnostics: (i) high-speed imaging with spectral filtering to resolve species spatiotemporal evolution; (ii) triple Langmuir probe scans to map *n_e_* and *T_e_* distributions in two orthogonal planes; (iii) XPS to characterize angular-dependent film composition and bonding; (iv) SPM to quantify surface roughness; and (v) UV-Vis spectrophotometry to measure optical transmittance and reflectance. By correlating these datasets, we establish direct quantitative links between plasma anisotropy and film chemo-optical anisotropy. Beyond providing critical data for contamination risk assessment and microsatellite component protection, this work offers a transferable framework for optimizing other plasma deposition processes, such as plasma-enhanced chemical vapor deposition (PECVD) of fluorocarbon films and DLC films. The plasma diagnostics and ion–neutral competition framework developed here may also offer methodological insights for laser-based transfer processes such as laser-induced forward transfer (LIFT) and laser-induced backward transfer (LIBT).

## 2. Experimental Setup

### 2.1. Vacuum Chamber and APPT Configuration

Experiments were conducted in a cylindrical vacuum chamber (2.5 m diameter, 3.0 m length) pumped by a two-stage vacuum system to a base pressure of 3 × 10^−3^ Pa. The pre-evacuation stage consists of one ZJP-600 Roots pump (Zhejiang Vacuum Pump Co., Ltd., Wenling, China) with an overflow valve and two 2X-70A oil-sealed rotary vane pumps (Shanghai Vacuum Pump Factory Co., Ltd., Shanghai, China), which reduce the chamber pressure to 5 Pa. The main pumping stage then comprises one 2X-30A oil-sealed rotary vane pump (Shanghai Xinnuo Instrument Group Co., Ltd., Shanghai, China), one Z-300 oil diffusion booster pump (Liaoning Zhenlong Vacuum Equipment Manufacturing Co., Ltd., Shenyang, China), and two K-900C oil diffusion pumps (Kunhong Vacuum Technology Co., Ltd., Tai’an, China), which further evacuate the chamber to the APPT operating pressure of 3 × 10^−3^ Pa.

The APPT was equipped with parallel oxygen-free copper electrodes (35 × 15 × 4 mm). Four parallel 3 μF capacitors were charged to 1500 V, delivering a discharge energy of 13.5 J per pulse. The thruster operated at a repetition frequency of 1 Hz. A cubic PTFE block was used as the propellant.

### 2.2. Contamination Deposition Sampling

Based on prior findings [25,26,27,28], plume contamination was considered negligible beyond ±50° in the electrode-parallel plane and beyond ±90° in the electrode-perpendicular plane. Therefore, substrates were placed at 15 cm from the PTFE surface, at 15° intervals from 0° to ±90° in the electrode-perpendicular plane and from 0° to ±45° in the electrode-parallel plane (Figure 1).

Optical glass substrates (20 × 20 × 1 mm) were sequentially ultrasonically cleaned in acetone, absolute ethanol, and deionized water (electrical conductivity ≤0.01 mS/m at 25 °C, 20 min each), then dried at 70 °C prior to deposition. The APPT was operated for 15 h (54,000 pulses at 1 Hz) to ensure sufficient film thickness for characterization.

### 2.3. Thruster Performance

The APPT discharge current was measured using a CWT150 Rogowski coil (Power Electronic Measurements Ltd., Nottingham, UK) and recorded using a Tektronix DPO4034 oscilloscope (350 MHz bandwidth, 2.5 GSa/s; Tektronix, Inc., Beaverton, OR, USA). Impulse bit was determined with a calibrated torsional pendulum (resolution 0.5 µN·s, uncertainty 2%) [29]. The total PTFE mass loss over 54,000 pulses was measured with a Mettler Toledo XS205DU microbalance (dual-range: 81 g/220 g, readability: 0.01 mg/0.1 mg; Mettler Toledo, Greifensee, Switzerland). The ablated mass per pulse was then calculated as the total mass loss divided by the number of pulses.

### 2.4. Plasma Diagnostics

High-speed imaging (XXRapidFrame, Stanford Computer OpticsInc., Berkeley, CA, USA) captured plasma evolution at 0.5 µs intervals from 0.1 µs to 7.6 µs after discharge onset. This time window covers all key discharge phases (current sheet formation, magnetic pinch, re-striking, and neutral expansion) that influence plume contamination and film deposition.

Narrowband filters were mounted in front of the camera lens to isolate specific species: a 430 nm filter for C^+^ ions and a 515 nm filter for C_2_ neutral molecules [12]. Exposure times were 10 ns for unfiltered imaging (total emission), 40 ns for the 430 nm filter, and 20 ns for the 515 nm filter. Grayscale images were pseudo-colored to enhance morphological features and relative intensity.

**Figure 2:**
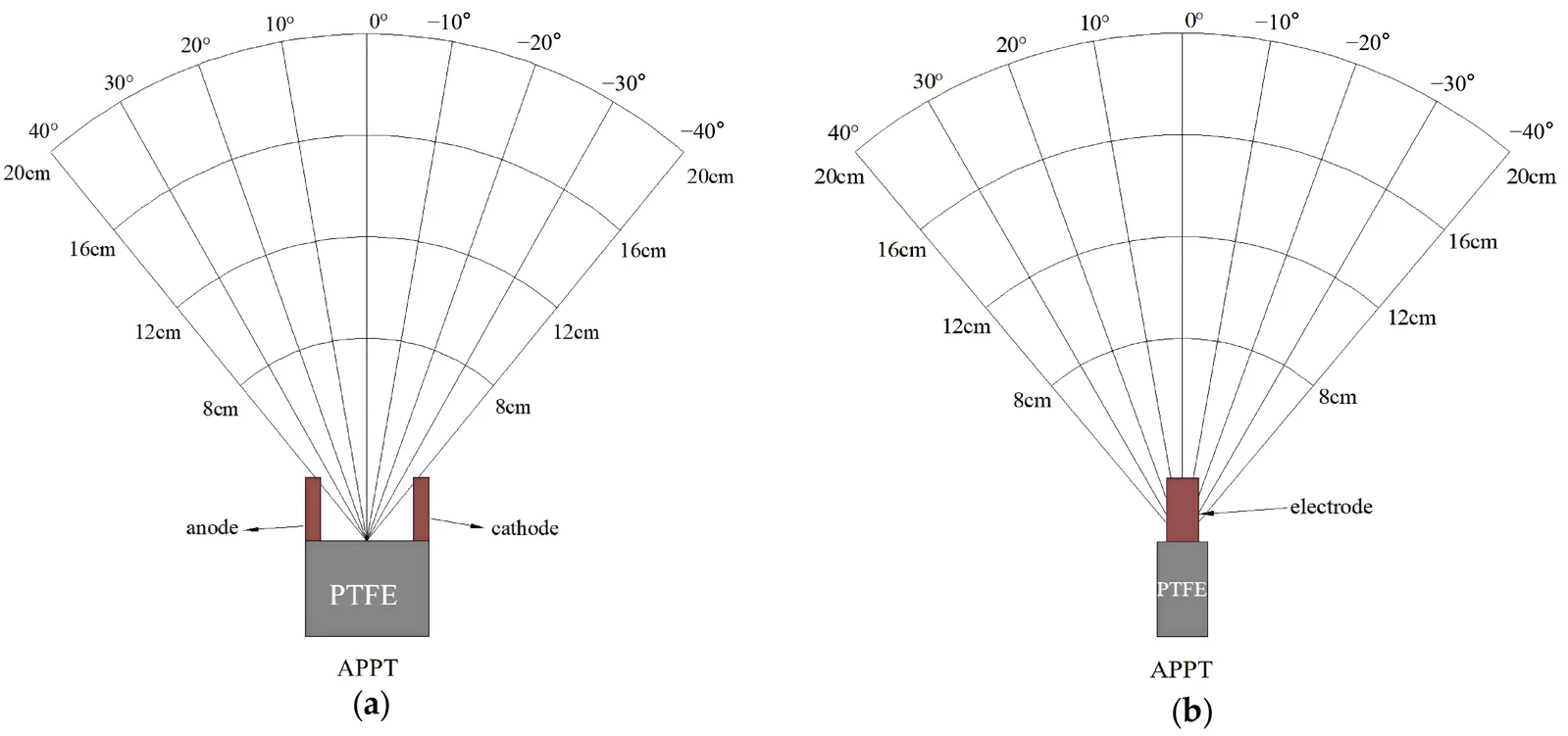
Schematic of the triple Langmuir probe diagnostic setup in (**a**) the electrode-perpendicular and (**b**) the electrode-parallel planes of the APPT.

A current-mode triple Langmuir probe system consisted of three tungsten wires (each 0.2 mm in diameter, 5 mm exposed length) arranged in an equilateral triangle with 1.0 mm spacing. The measurement principle is described in Ref. [10]. The system measured *n_e_* and *T_e_* in both planes over angles from −40° to +40° and radial distances of 8 to 20 cm (Figure 2).

At each grid point, data from 10 consecutive discharge cycles were averaged. Estimated uncertainties: ±15% for *T_e_*, ±10% for *n_e_*. The probe was cleaned by electron bombardment after every 25 discharges to remove fluorocarbon deposits [29,30].

Because *n_e_* in the APPT plume spans approximately two orders of magnitude (from 10^19^ to 10^21^ m^−3^), the *n_e_* data were log-transformed for contour plotting, with linear interpolation between grid points.

### 2.5. XPS Analysis of Film Composition and Chemical Bonds

XPS analysis was conducted using a Thermo Fisher Scientific K-Alpha 1063 spectrometer (Thermo Fisher Scientific, Waltham, MA, USA) equipped with a monochromatic Al Kα source (72 W, 400 μm spot size). Both survey spectra and high-resolution C 1s spectra were acquired for each deposited film. To minimize microscale compositional inhomogeneity, three measurements at distinct locations (~1 mm apart) on each substrate were averaged.

Binding energies were calibrated against the C-C peak at 284.7 eV to compensate for surface charging. No sputtering was applied prior to analysis to preserve native fluorine content. High-resolution C 1s spectra were acquired because this core-level transition produces the most distinct chemical shifts for differentiating fluorocarbon bonding states, while the F 1s peak is less sensitive to subtle changes in the local chemical environment [31]. Data processing was performed using Avantage v5.9931 (Thermo Fisher Scientific, Waltham, MA, USA). High-resolution C 1s spectra were deconvoluted using a Shirley background and a Gaussian–Lorentzian line shape. Atomic concentrations were calculated using the relative sensitivity factor method via the following equation [23,30]:

(1) $$c_{k}=\frac{A_{k}/S_{k}}{\sum_{j}A_{j}/S_{j}},$$

where *c_k_* is the atomic concentration of element *k*, *A_k_* is the XPS peak area of element *k*, and *S_k_* is the sensitivity factor of element *k*. The sensitivity factor values for F, C, O, Cu, and N are 4.43, 1.00, 2.93, 25.39, and 1.8, respectively.

The bonded fluorine-to-carbon ratio *F*/*C* was calculated via:

(2) $$F/C=\frac{A_{F}/S_{F}}{A_{C}/S_{C}},$$

Reproducibility was assessed by standard deviations from triplicate measurements; uncertainties are shown as error bars in the corresponding figures. All reported compositional and bonding values are based on averaged spectra and the peak fitting procedure described above.

### 2.6. Surface Morphology Characterization

Surface morphology was characterized using scanning probe microscopy (SPM, NT-MDT TS-150, NT-MDT Spectrum Instruments, Moscow, Russia) in tapping mode over a 50 × 50 μm^2^ scan area. Root-mean-square roughness (*R_q_*) was averaged from four randomly selected regions per sample. The *R_q_* of the bare glass substrate (7.9 nm) served as a baseline. Morphological observations, combined with quantitative roughness analysis, provide insights into the competing effects of ion bombardment and neutral particle deposition during film growth, and help interpret the optical properties discussed in Section 3.7.

### 2.7. Film Thickness and Chemo-Optical Property Characterization

Film thickness was determined from cross-sectional scanning electron microscopy (SEM, Hitachi S-4800, Hitachi High-Technologies Corporation, Tokyo, Japan). Samples were sputter-coated with a thin gold layer to avoid charging. For each angular position, five measurements were taken at different cross-section locations, and the average was reported.

Optical transmittance and specular reflectance spectra were acquired over the wavelength range of 300–1500 nm using a Hitachi U-4100 UV-Vis-NIR double-beam spectrophotometer (Hitachi High-Technologies Corporation, Tokyo, Japan). Transmittance was measured under normal-incidence (0°) conditions with the film-coated side facing the incident beam, and a baseline scan using air as the reference was conducted before sample measurement. Specular reflectance was collected with a 5° absolute reflectance accessory, where the incident beam illuminated the film-coated sample surface at 5° relative to the surface normal; absolute reflectance values were calibrated employing the standard aluminum reference mirror provided with the instrument.

All spectra were recorded at 1 nm intervals with a scan speed of 120 nm/min, and each spectrum was averaged from three consecutive scans to improve signal-to-noise ratio. Bare glass substrates from the same batch were measured under identical conditions as references.

## 3. Results and Discussion

### 3.1. APPT Discharge Dynamics and Performance Characteristics

**Figure 3:**
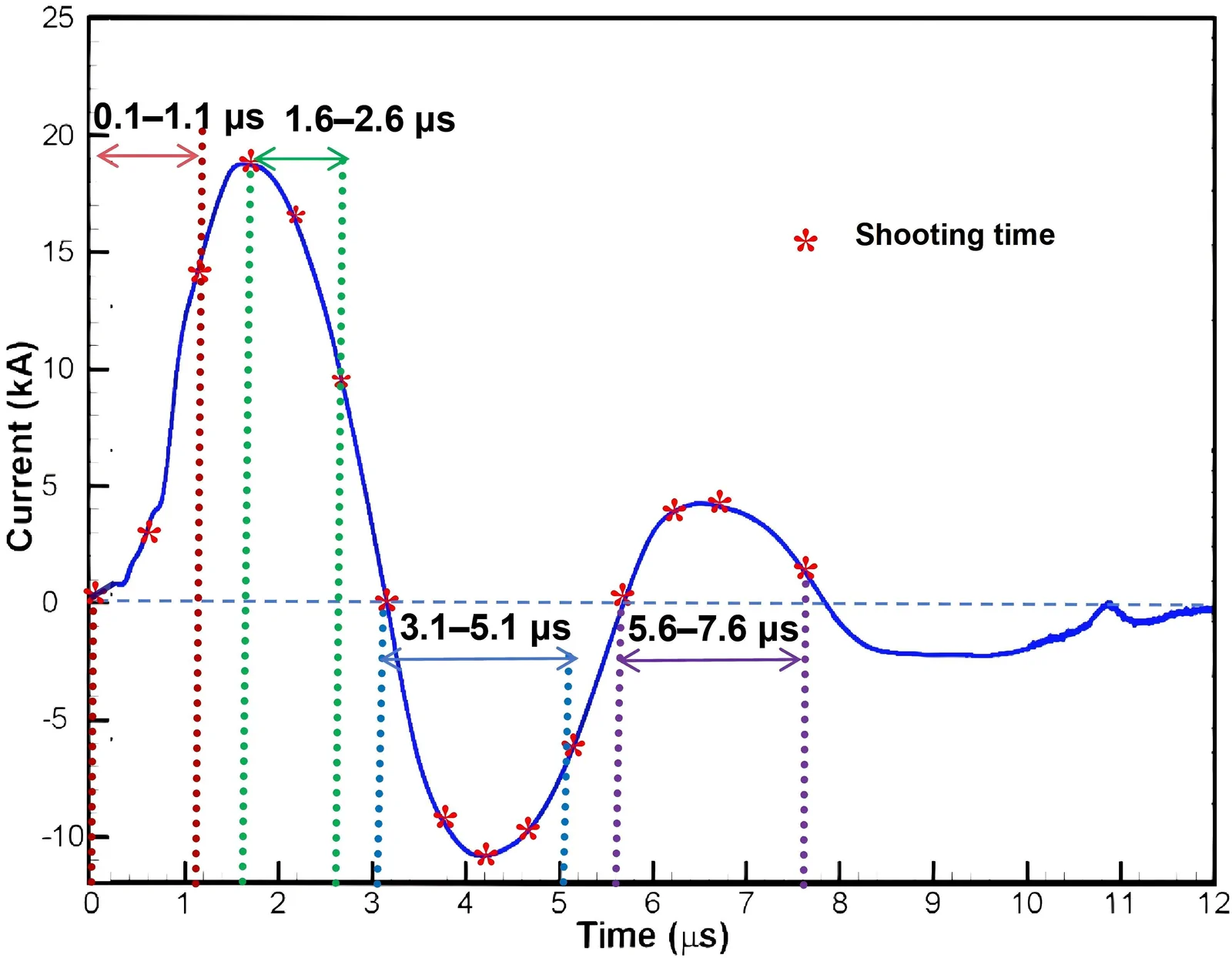
Timing of high-speed image acquisition relative to the discharge current waveform.

At a discharge energy of 13.5 J per pulse, the APPT ablated 41.94 ± 0.2 μg of PTFE per pulse and produced an impulse bit of 208.5 ± 0.3 μN·s. To elucidate the plasma dynamic mechanisms governing film deposition, high-speed imaging was synchronized with the discharge current waveform, capturing the plasma evolution from 0.1 μs to 7.6 μs after discharge initiation (Figure 3). The acquisition timing is marked by vertical dashed lines overlaid on the current waveform, enabling direct correlation between plasma morphology and instantaneous current. This temporal window covers all key discharge phases that directly influence plume contamination and film deposition.

**Figure 4:**
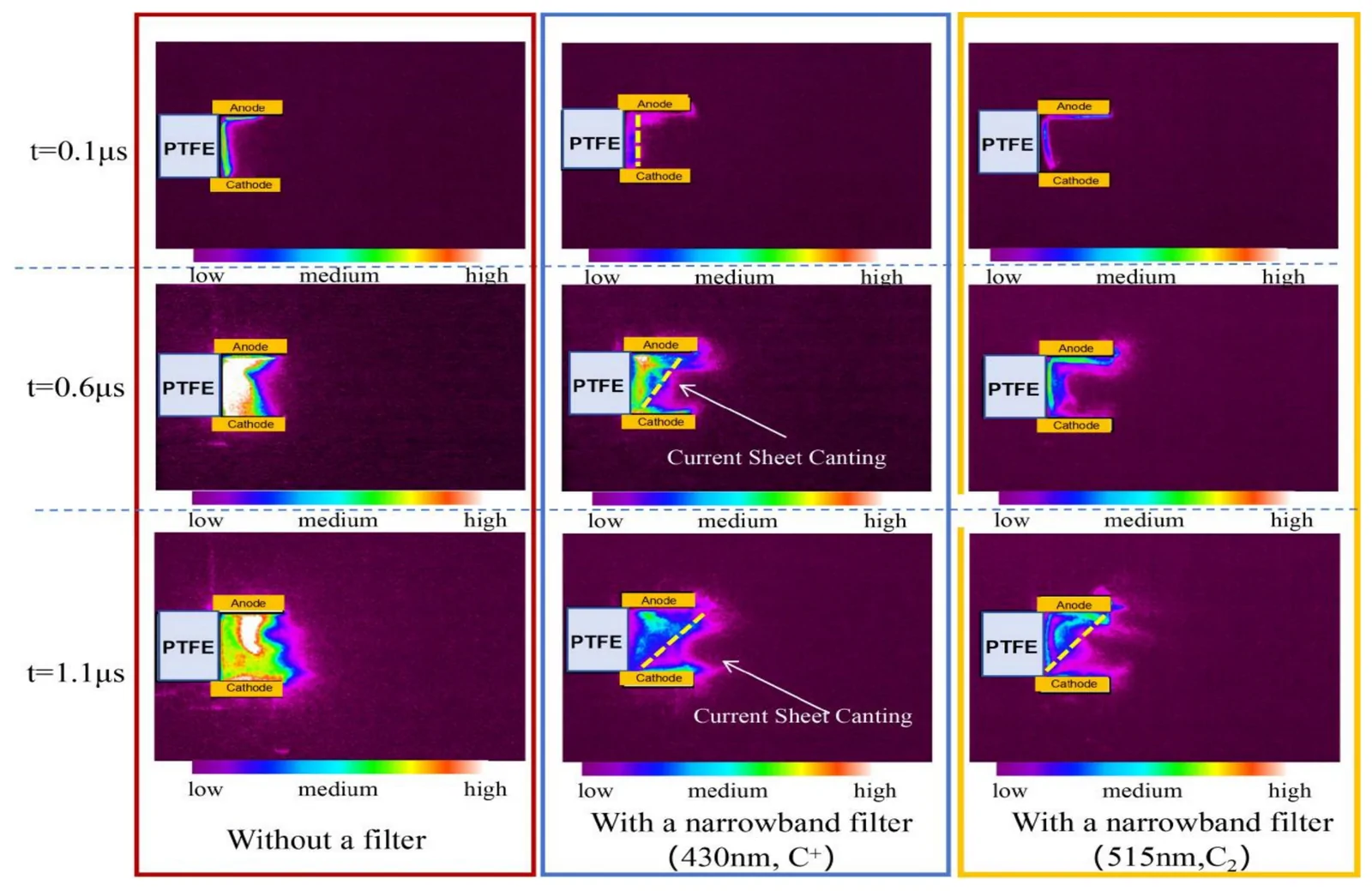
Formation and canting of the initial current sheet.

Initial current sheet formation and canting (0.1–1.1 μs): Figure 4 shows a constricted discharge channel forming across the PTFE surface within 0.1 μs of discharge onset. As the discharge current increases, plasma luminosity intensifies, and the ionized material expands axially under the Lorentz force. Notably, the arc deviates from an ideal planar current sheet, exhibiting current sheet canting (the tilting of the current sheet from an ideal planar geometry)—an inclination attributed to plasma non-equilibrium and the Hall effect [26,32]. This earliest phase establishes the fundamental initial condition for the anisotropic plasma distribution that persists throughout the discharge.

**Figure 5:**
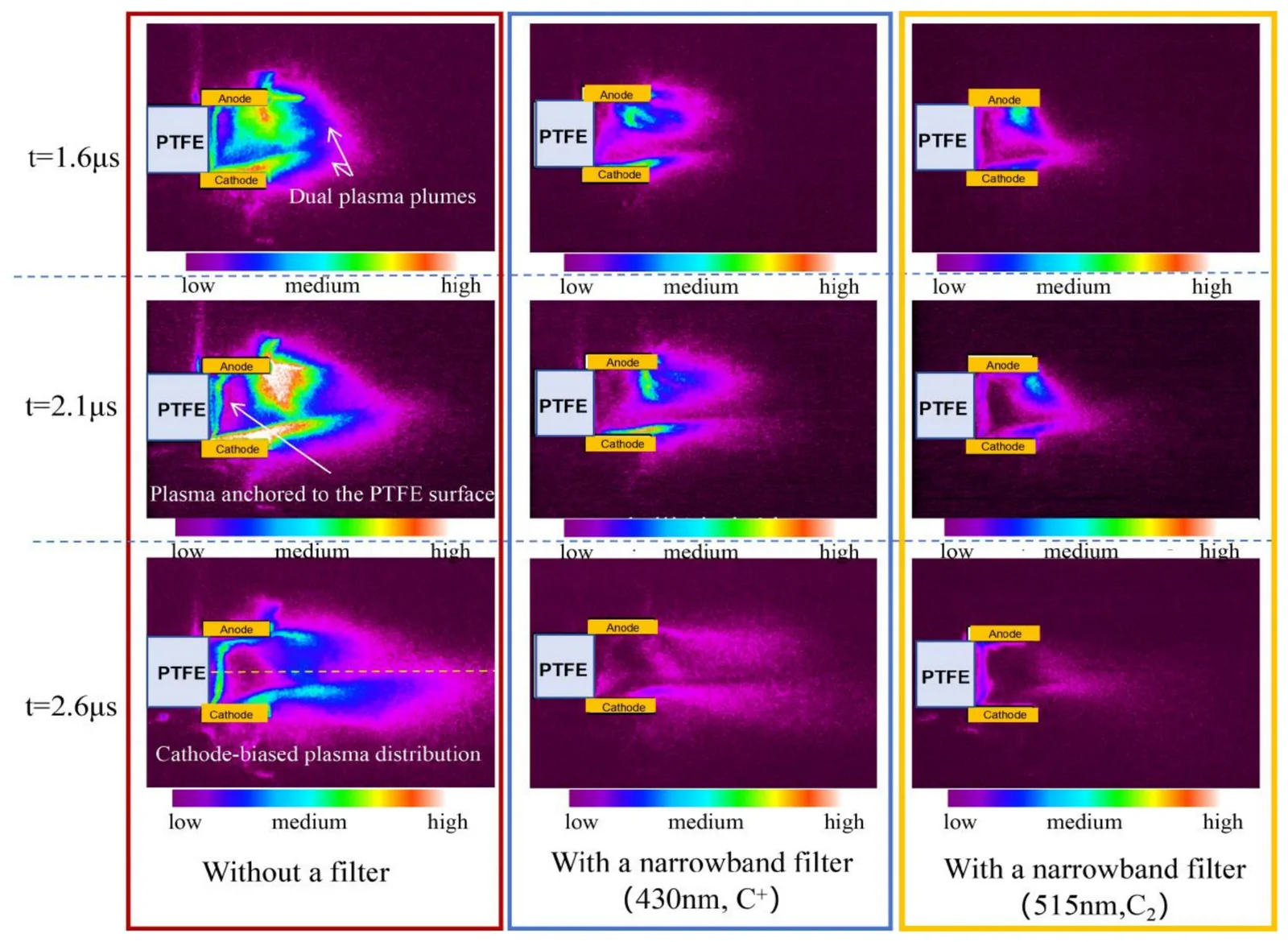
Magnetic pinch effect and development of asymmetric plasmas.

Magnetic pinch and asymmetric plume development (1.6–2.6 μs): As illustrated in Figure 5, the intense discharge current interacts with its self-generated magnetic field, producing a pinch force that compresses the arc radially. Near the electrodes, the current density—and thus the induced magnetic field strength—is higher than in the central region, creating a pressure gradient that drives plasma toward the arc center [33,34].

Consistent with this mechanism, magnetic pinch becomes prominent at 1.6 μs, generating dual plasma plumes from the electrode roots. Time-resolved C^+^ imaging reveals a persistent velocity asymmetry: cathode-side plasma propagates faster and decays more slowly than its anode-side counterparts. This cathode-biased plasma distribution characteristic is corroborated by Langmuir probe measurements (Section 3.2), which directly quantify the anisotropic electron density distribution in the plume.

Throughout this interval, the plasma arc remains anchored to the PTFE surface. sustaining PTFE thermal decomposition via conductive heat transfer. Meanwhile, surface discharge channels divert capacitor energy, reducing the energy available for plasma acceleration. A distinct dark region appears in the C^+^ imaging at 2.6 μs, marking the onset of plasma ionization attenuation as the discharge current decreases.

**Figure 6:**
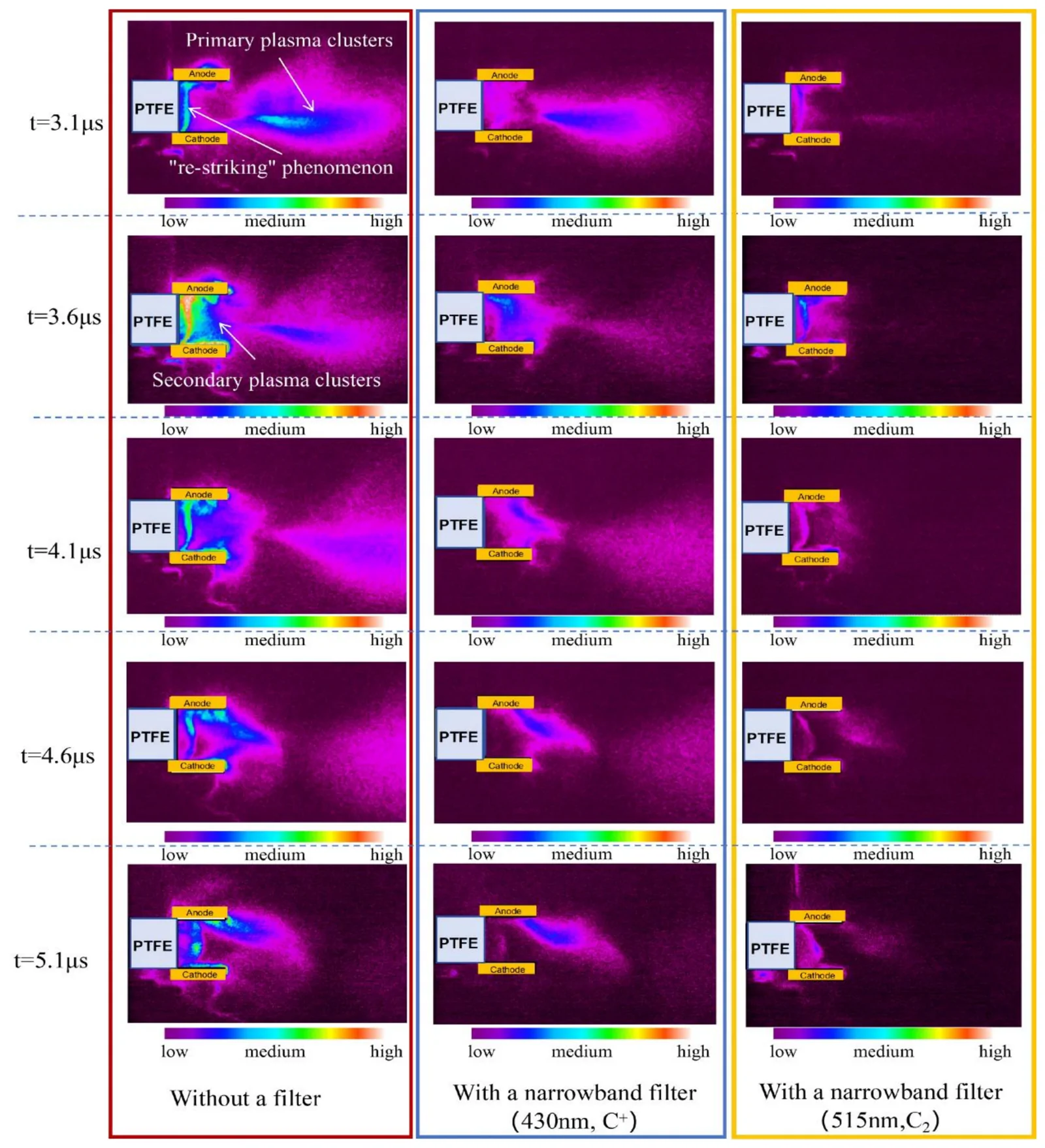
Secondary plasma clusters generated by reverse charging and re-striking.

Reverse charging and secondary plasma formation (3.1–5.1 μs): As shown in Figure 6, capacitor reverse charging triggers flashover discharges across the PTFE surface—a phenomenon known as re-striking [35,36]. The residual capacitor energy generates secondary plasma clusters with significantly lower ionization levels. Meanwhile, the reversed discharge current reduces C^+^ emission intensity, while low-velocity neutral plasma clusters accumulate near the PTFE ablation surface.

**Figure 7:**
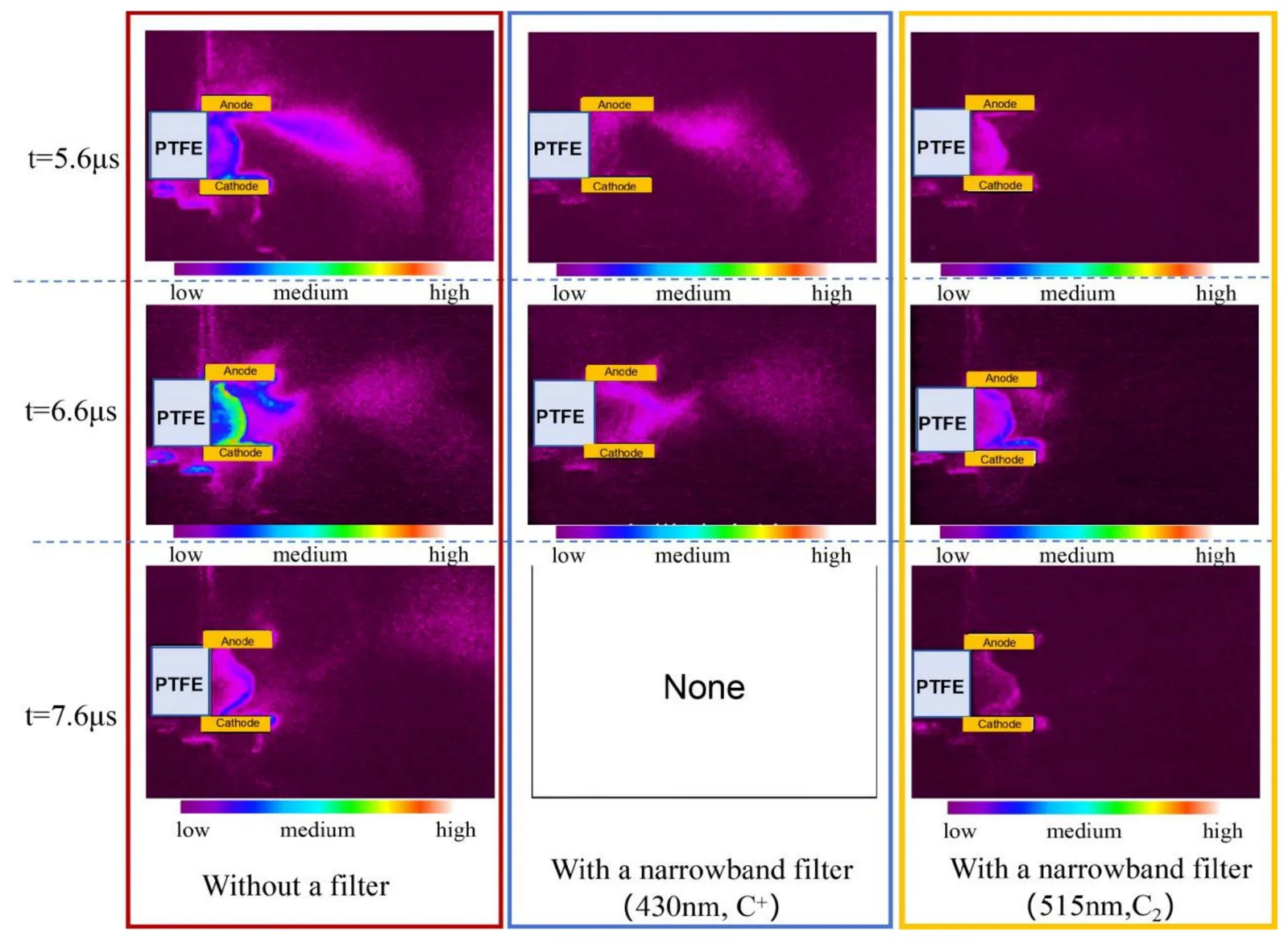
Neutral-dominated expansion in the final discharge phase.

Neutral-Dominated Expansion (5.6–7.6 μs): After 5.6 μs (Figure 7), the discharge current and energy drop below the threshold required for sustaining significant plasma ionization, and the C^+^ emission signal falls below the detection limit. In contrast, neutral plasma clusters continue to expand toward the nozzle under gas-dynamic forces, remaining detectable until 7.6 μs.

Overall, high-speed imaging resolves four sequential discharge phases that establish the spatiotemporal foundation for the asymmetric plasma distribution in the APPT plume. The key observations—Hall-effect-induced current sheet canting, cathode-biased plasma velocity asymmetry, and persistent neutral expansion—provide a qualitative basis for understanding the asymmetric plasma parameter distribution (Section 3.2) and the subsequent spatial variation in film composition (Section 3.3 and Section 3.4).

### 3.2. Spatiotemporal Distribution of Plasma Parameters

**Figure 8:**
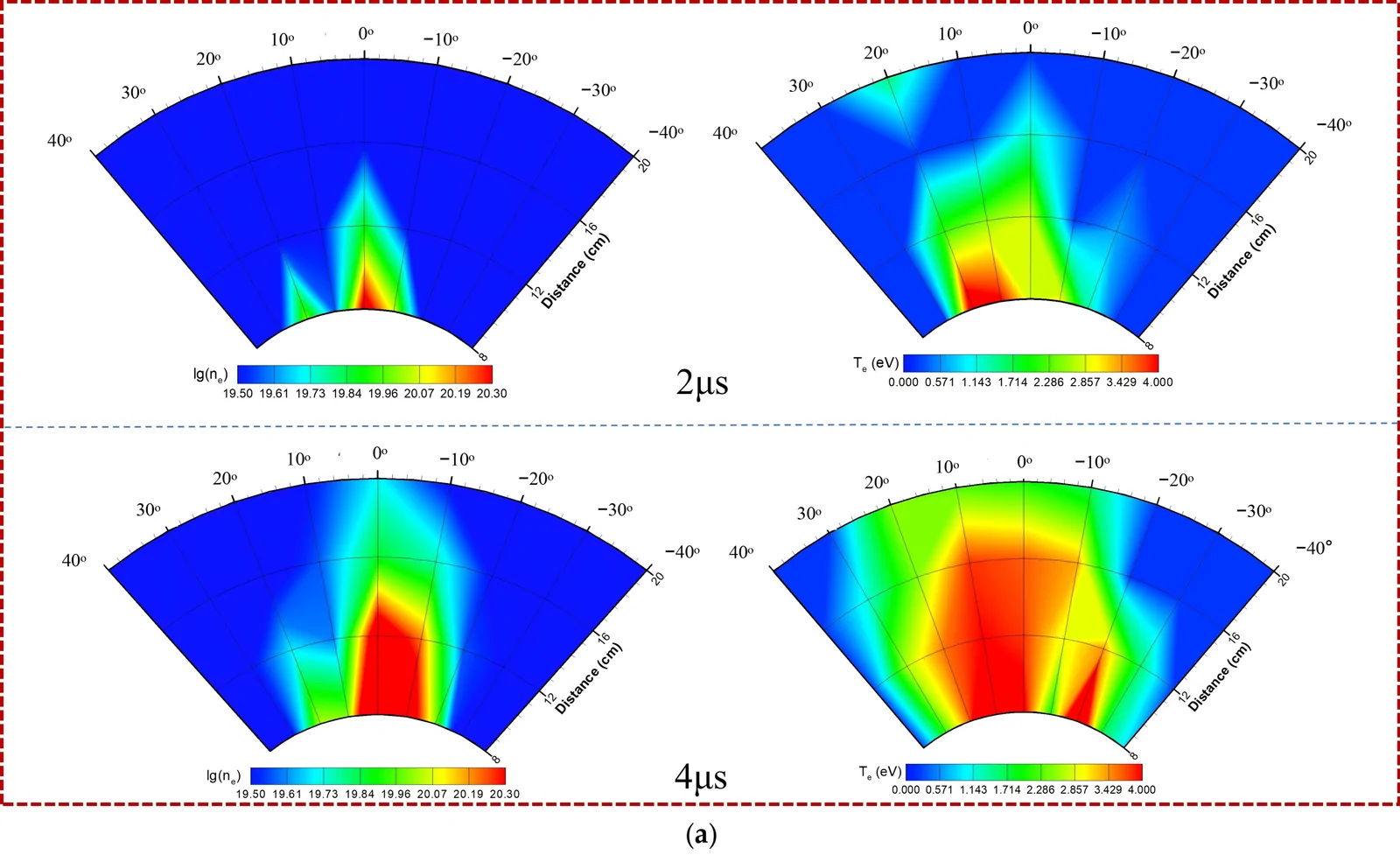

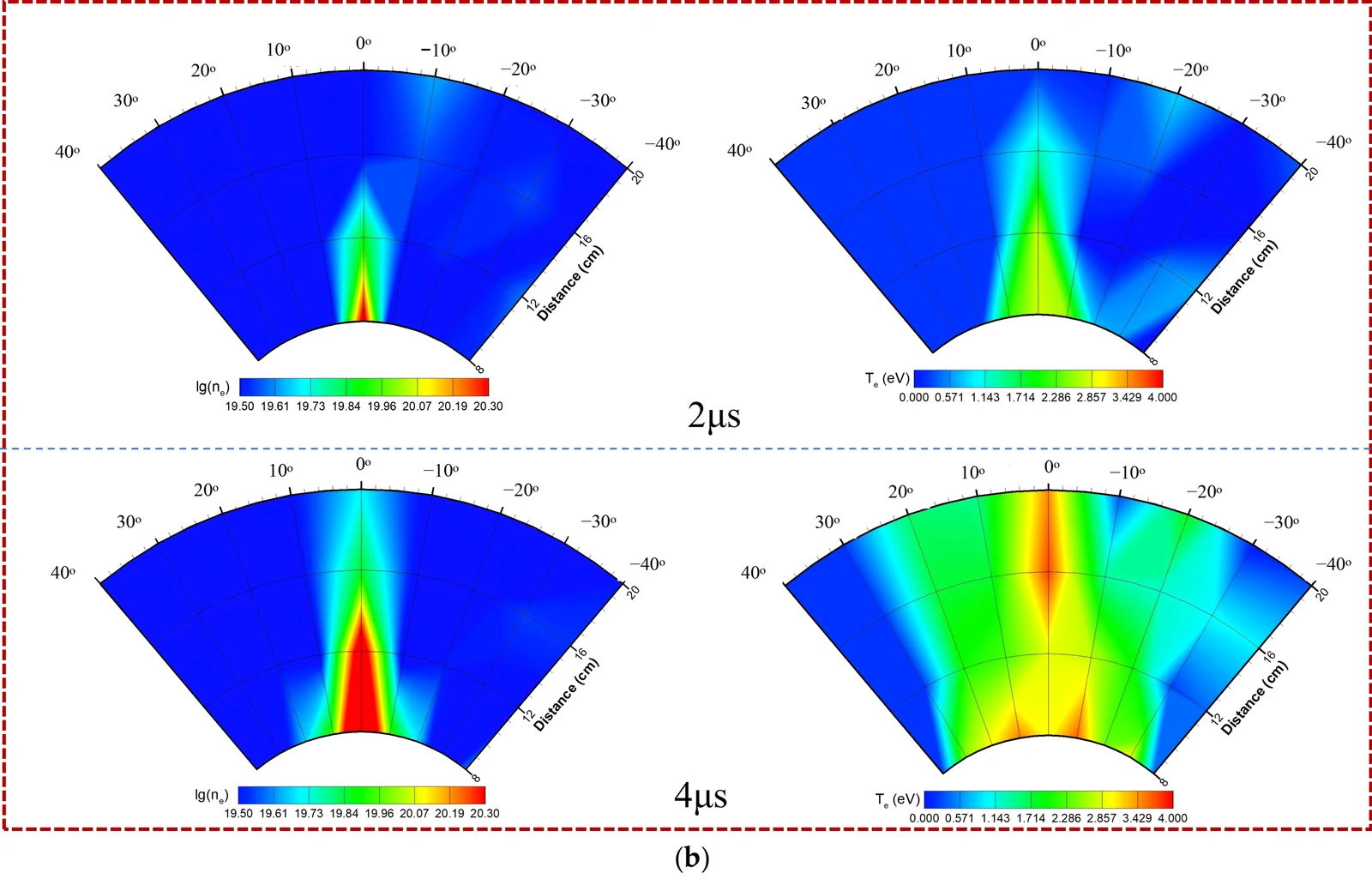
Spatiotemporal distributions of electron temperature and number density. (**a**) Electrode-perpendicular plane. (**b**) Electrode-parallel plane.

The asymmetric plasma dynamics observed in Section 3.1—particularly the cathode-biased velocity and intensity—are directly reflected in the spatial distribution of *n_e_* and *T_e_*. Figure 8 maps these distributions at 2 μs and 4 μs after discharge, providing quantitative insight into the plasma environment that governs film deposition heterogeneity.

In the electrode-perpendicular plane (Figure 8a), *n_e_* is cathode-biased, decaying more slowly with angle and distance on the cathode side than on the anode side. Peak *n_e_* reaches approximately 8 × 10^20^ m^–3^ near the cathode side within the 0–10° angular range, while peak *T_e_* attains about 5 eV near the anode side. Both parameters decrease monotonically with increasing angle and radial distance. These observations agree with the cathode-biased plasma behavior seen in high-speed imaging (Section 3.1) and prior reports [25,27], and are attributed to the combined Hall effect and magnetic pinch.

The Hall effect induces electron drift perpendicular to both electric and magnetic fields, tilting the current sheet and generating a lateral force that preferentially accelerates plasma toward the cathode [32]. Magnetic pinch then amplifies this asymmetry by confining higher-density plasma near the electrode roots, where higher current density reinforces cathode-side plasma retention [29].

Concurrently, *T_e_* exhibits a pronounced anode-side bias. This originates from the same Hall-effect-induced current sheet dynamics: the skewed discharge current causes earlier arc attachment on the anode, where concentrated current density intensifies Joule heating, elevating *T_e_* relative to the cathode side [29,32]. This non-uniform distribution of electron number density and temperature directly governs the asymmetric flux of energetic ions incident on substrates—a key factor influencing film composition and growth rate. Specifically, higher ion flux on the cathode side promotes preferential fluorine sputtering, leading to lower *F*/*C* values in the deposited films [23,24].

In contrast, the electrode-parallel plane (Figure 8b) displays axial symmetry. High-*n_e_* regions are confined within ±10° of the central axis. Peak *n_e_* near the axis is comparable to that in the perpendicular plane, but decays more rapidly with angle, dropping by an order of magnitude beyond ±10°. *T_e_* remains relatively uniform within ±10° and decreases slowly at larger angles. This symmetry arises from the uniform electrode geometry, which eliminates polarity-driven asymmetries and balances Hall-effect-induced lateral forces across the central axis. Unlike in the perpendicular plane, the azimuthal magnetic field in the parallel plane is uniform, and the symmetric magnetic pinch around the central axis maintains axial symmetry.

Under quasi-neutrality, the ion density follows the same spatial variation trends as the electron number density. Previous time-of-flight measurements have shown that ion velocity exhibits a similar angular dependence, decreasing with the increase in azimuthal angle and radial distance from the thruster exit [29]. Thus, *n_e_* distribution serves as a reliable proxy for relative ion flux: higher *n_e_* indicates stronger energetic ion bombardment on the substrate.

In summary, the plasma parameters exhibit distinct planar characteristics: in the perpendicular plane, *n_e_* is biased toward the cathode while *T_e_* is biased toward the anode; in the parallel plane, the distribution is axially symmetric. This heterogeneity in *n_e_* and plasma velocity governs the energetic ion flux, creating distinct deposition environments in the two orthogonal planes. By quantifying the asymmetry first observed in Section 3.1, these findings provide a mechanistic basis for interpreting the planar differences in film composition, thickness, surface morphology, and optical properties.

### 3.3. Angular Dependence of Film Composition and Chemical Bond Configurations

**Figure 9:**
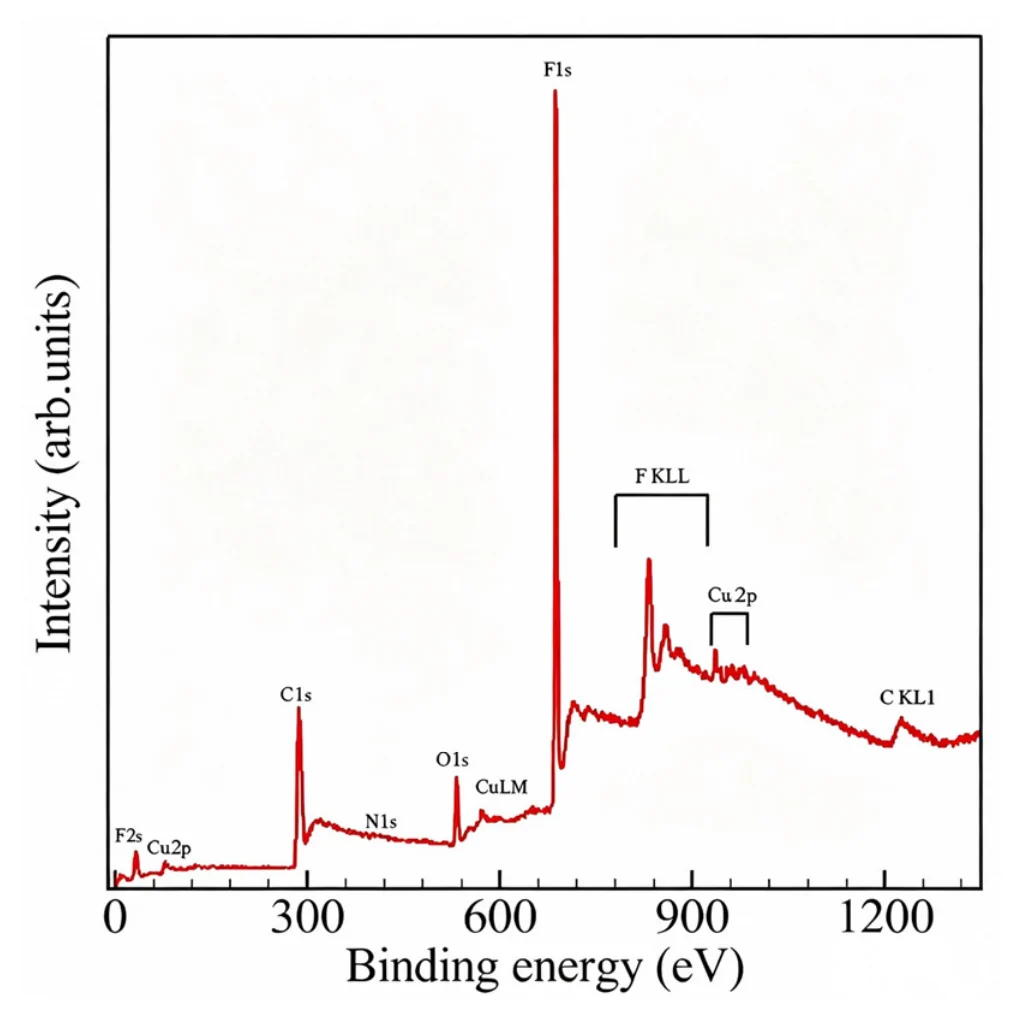
Representative XPS survey spectrum of a fluorocarbon film deposited at 0°.

A representative XPS survey spectrum of a film deposited at 0° is shown in Figure 9. Five elements are clearly identified: carbon (C 1s, 284.7 eV), fluorine (F 1s, 690 eV), oxygen (O 1s, 532 eV), copper (Cu 2p, 933 eV), and nitrogen (N 1s, 399.75 eV).

**Figure 10:**
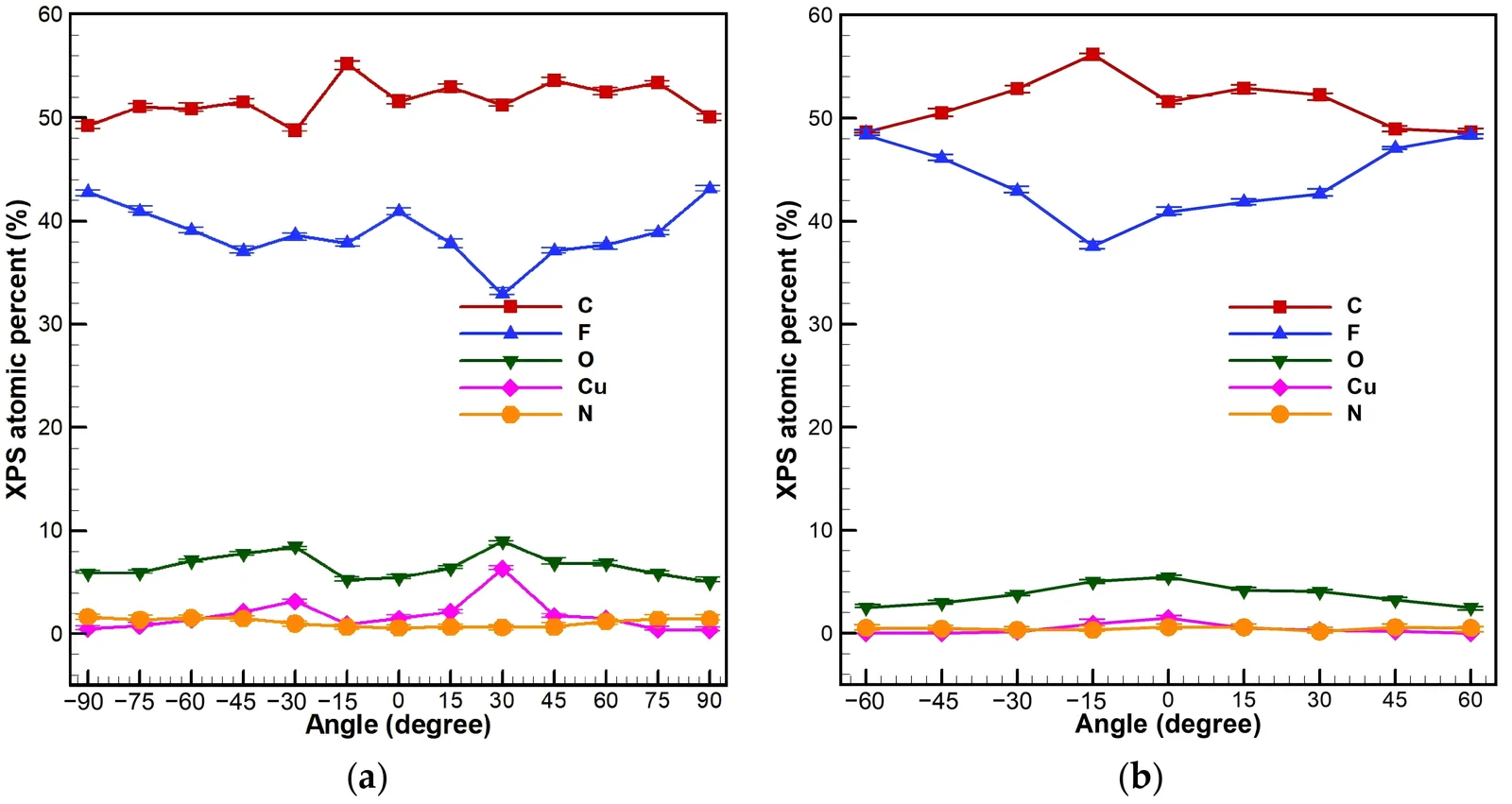
Angular dependence of XPS elemental composition of deposited films: (**a**) electrode-perpendicular plane and (**b**) electrode-parallel plane.

Quantification using Equation (1) reveals a systematic angular dependence of elemental composition (Figure 10). Carbon (49–56 at.%) and fluorine (35–48 at.%) dominate, both derived from the ablation and ionization of the PTFE propellant, though the fluorine content is lower than that in pristine PTFE. Triplicate analyses per sample yielded SD ranges: C (0.5–1.0 at.%), F (0.4–0.9 at.%), O (0.1–0.2 at.%), N (0.1–0.2 at.%), and Cu (0.1–0.3 at.%).

Copper (0.5–2.3 at.%) originates from electrode erosion during discharge. Nitrogen (0.2–0.8 at.%) comes from the residual background gas in the vacuum chamber. Oxygen (0.2–0.8 at.%) is attributed to two main sources: (i) the reaction of unsaturated carbon bonds in the film with residual oxygen in the vacuum chamber during the deposition process; and (ii) the post-deposition oxidation of free carbon radicals upon air exposure.

High-resolution C 1s spectra were deconvoluted into five peaks: C-C/C-H (284.8 ± 0.1 eV), C-CF_x_/C-O (286.4 ± 0.4 eV), C-F (288.8 ± 0.4 eV), CF2 (291.1 ± 0.3 eV), and CF3 (293.2 ± 0.3 eV) [31,37].

**Figure 11:**
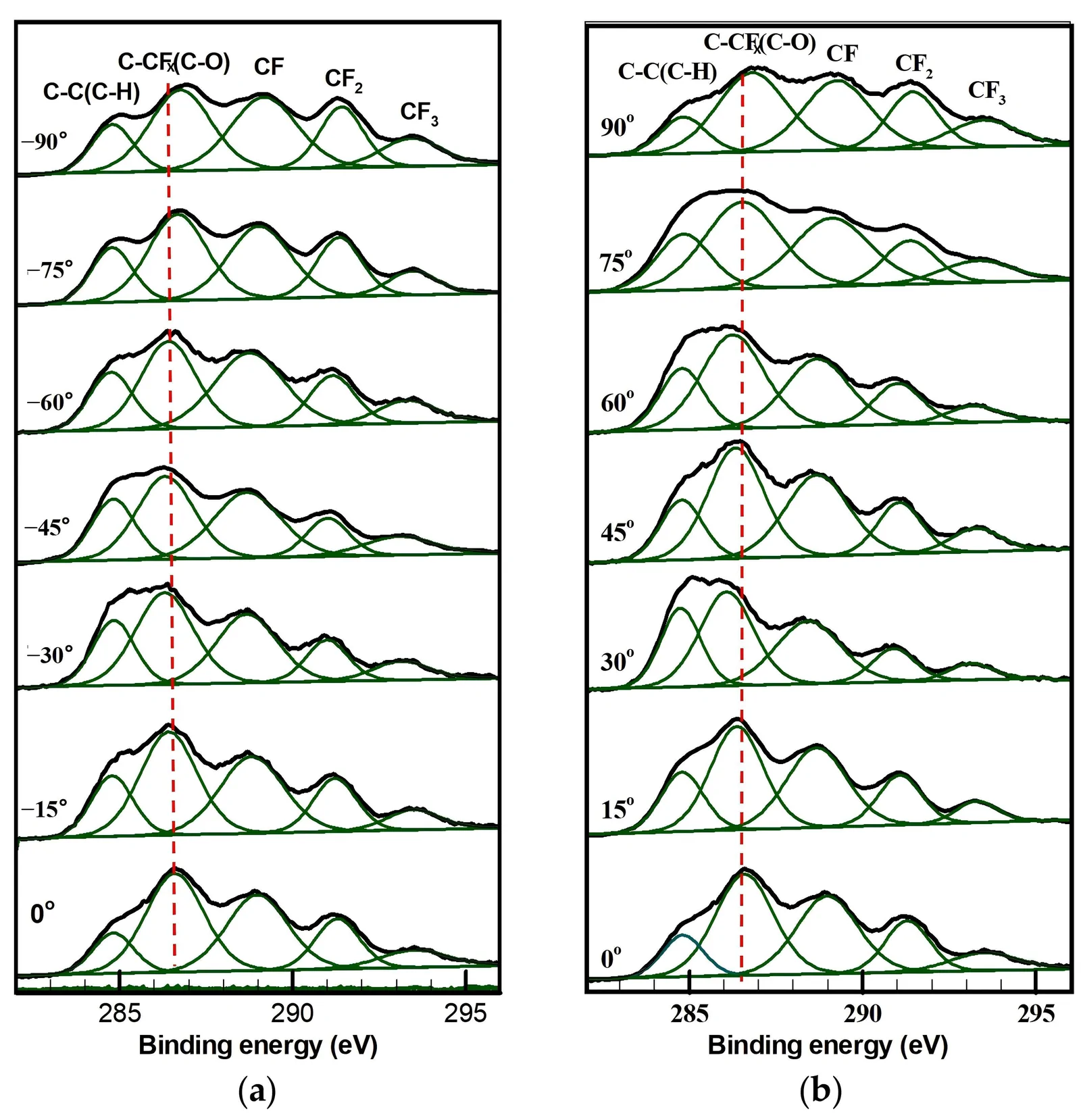

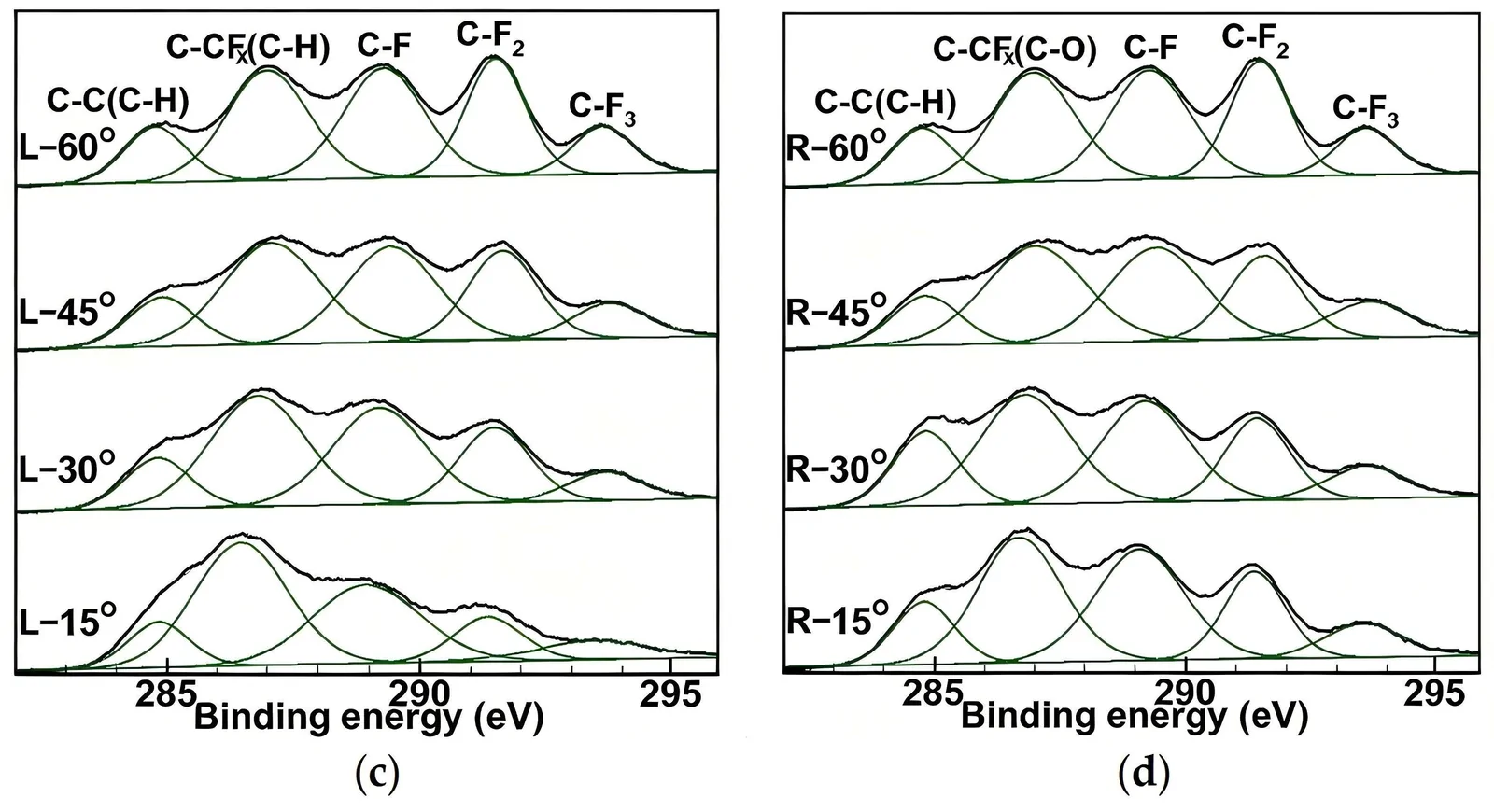
XPS C1s spectra and fitting results of APPT plume-deposited films: (**a**) electrode-perpendicular (cathode side), (**b**) electrode-perpendicular (anode side), (**c**) electrode-parallel (left side), (**d**) electrode-parallel (right side). The red dashed line marks the binding energy position of the C-CF_X_/C‑O component (286.4 ± 0.4 eV) for the film deposited at the 0° position.

Figure 11 shows the C 1s spectra and their corresponding fitting curves for films deposited at various spatial angles. As angle increases, binding energies shift differently in the two planes. In the electrode-perpendicular plane, both cathode- and anode-side bonds shift to lower energies from 0° to 30°, then to higher energies from 45° to 90°. In the electrode-parallel plane, binding energies consistently shift to higher values with increasing angle.

**Figure 12:**
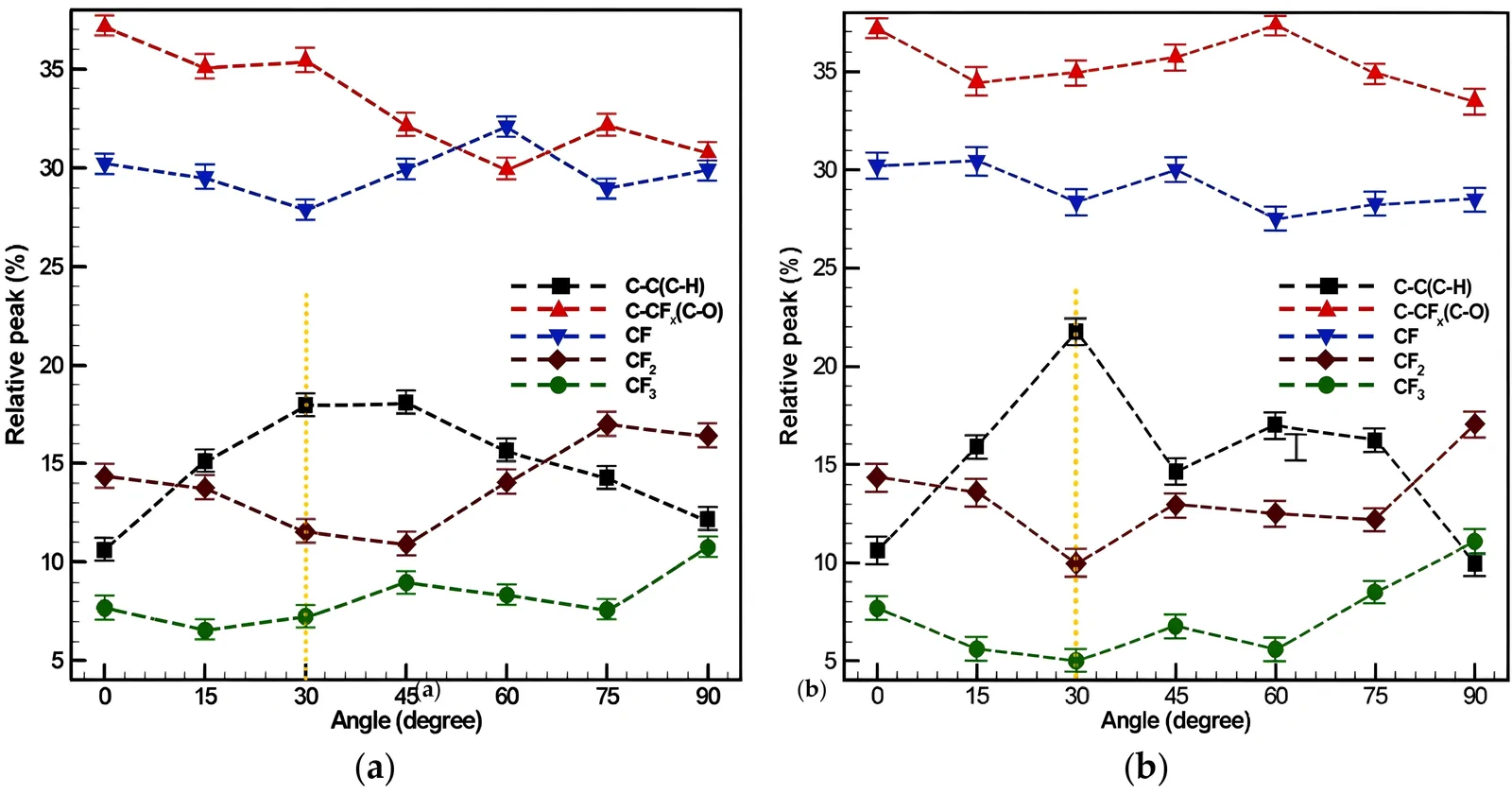
Angular dependence of chemical bond distribution of fluorocarbon films deposited on (**a**) cathode and (**b**) anode sides in the electrode-perpendicular plane. The yellow dashed line highlights the 30° location on both the cathode and anode sides for clear identification of the critical angle where the relative peak intensity changes.

**Figure 13:**
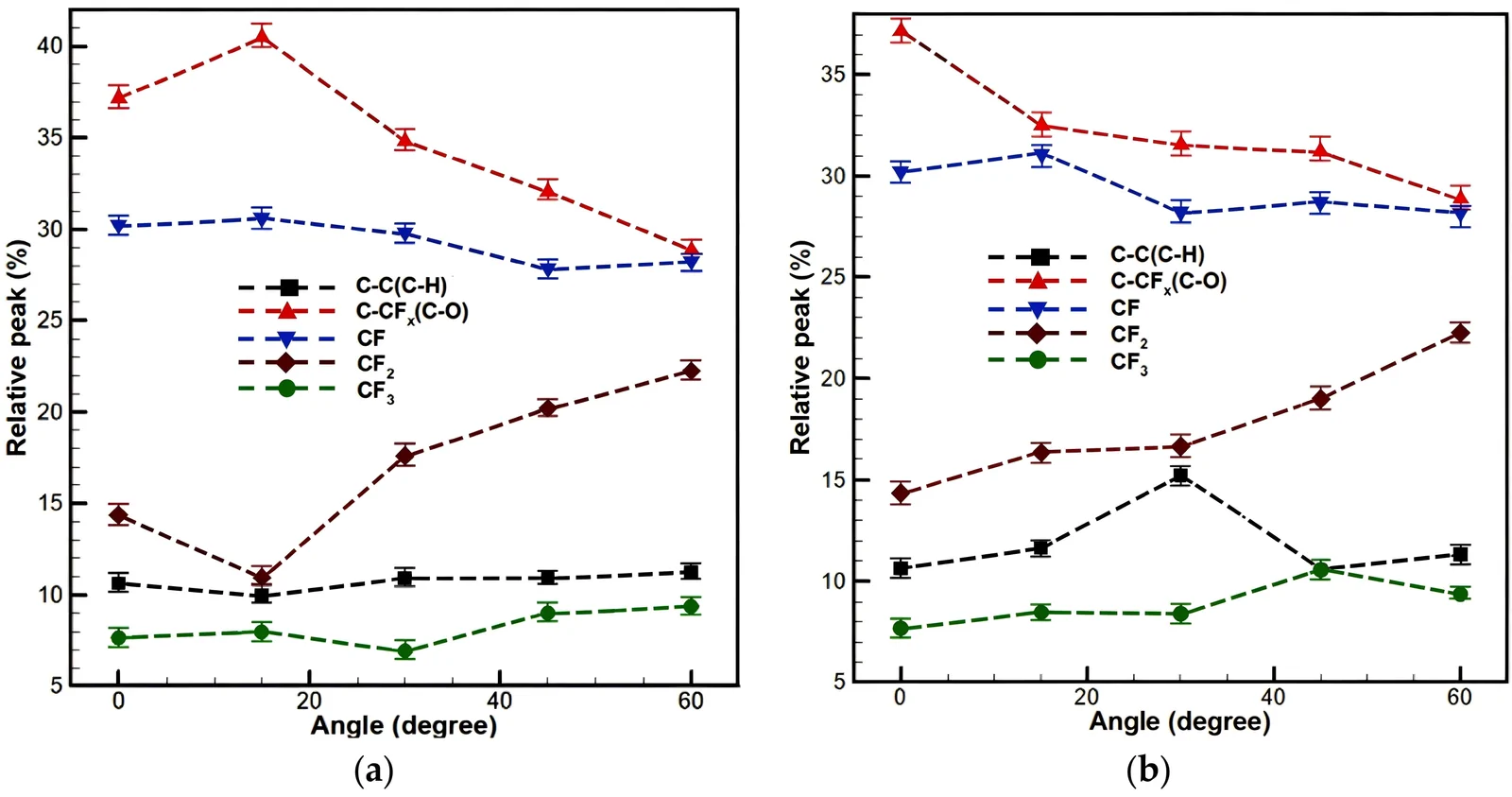
Angular dependence of chemical bond distribution of fluorocarbon films deposited on (**a**) left and (**b**) right sides in the electrode-parallel plane.

Angular-dependent relative peak intensities (Figure 12 and Figure 13) indicate that C-F and C-CF_x_/C-O bonds dominate, while CF_3_ bonds are least abundant. The reproducibility of the bond fraction measurements was verified via triplicate measurements per sample, with standard deviation (SD) ranges as follows: C-F (0.8–1.5%), C-CF_x_/C–O (0.6–1.3%), C-C/C-H (0.5–1.2%), CF_2_ (0.4–0.9%), and CF_3_ (0.3–0.8%).

The bonding composition shows distinct angle-dependent behaviors, which differ significantly between the two planes. In the electrode-perpendicular plane (Figure 12), on the cathode side, C-C/C-H and CF_2_ vary in opposite trends, with a transition near 45°: from 0° to 45°, C-C/C-H increases from 10.95% to 18.08% while CF2 decreases from 14.34% to 10.91%; beyond 45°, the trends reverse. On the anode side, a clear boundary appears at 30°: C-C/C-H peaks at 21.75% at 30° then declines, while CF_2_ follows the inverse pattern.

In the electrode-parallel plane (Figure 13), bond distributions are quasi-symmetric, consistent with plasma axial symmetry. As angle increases, C-CF_x_/C-O and C-F fractions decrease, while CF_2_ increases.

In summary, the chemical bonding configurations exhibit strong angular dependence with distinct planar trends. On the cathode side of the electrode-perpendicular plane, C–C/C–H and CF_2_ vary oppositely with a transition near 45°, while on the anode side a clear boundary appears at 30°. In the electrode-parallel plane, distributions are quasi-symmetric, with C–CF_x_/C–O and C–F decreasing and CF_2_ increasing with angle. These bonding characteristics provide the structural basis for *F*/*C* and optical property variations discussed next.

### 3.4. F/C Ratio of Deposited Films: Angular and Planar Effects

The plasma asymmetries established in Section 3.2 directly govern the flux and energy of species arriving at the substrate, thereby determining film composition and microstructure [37,38].

**Figure 14:**
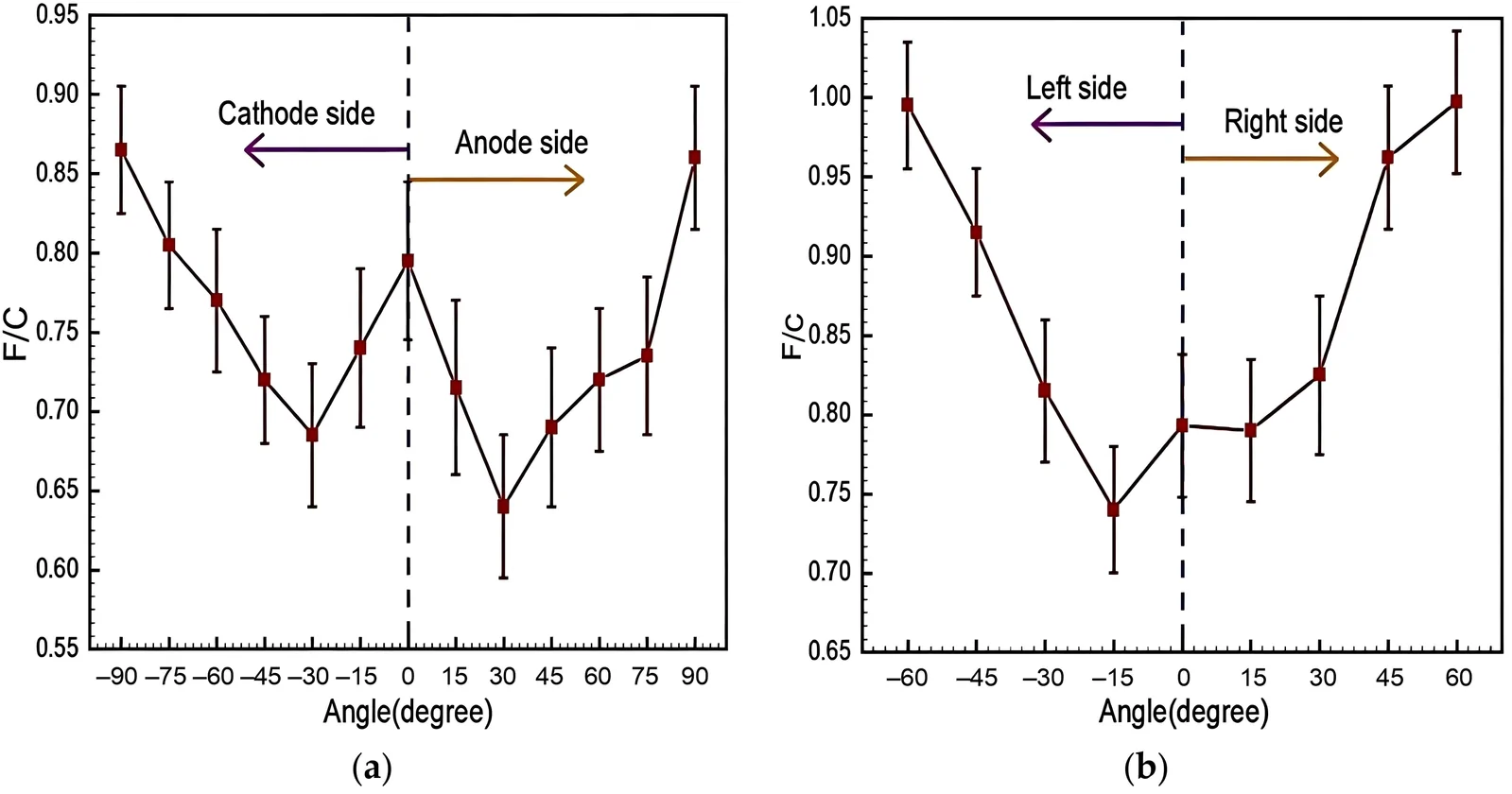
*F*/*C* of deposited films at various angles in (**a**) electrode-perpendicular and (**b**) electrode-parallel planes.

Figure 14 shows *F*/*C* values ranging from 0.6 to 1.0—significantly lower than those of pristine PTFE—with systematic angular dependence in both planes. Triplicate analysis gave an SD range of 0.04–0.06. This consistent fluorine deficiency arises from two competing growth processes: energetic ion bombardment, which preferentially sputters lighter fluorine atoms from the growing film, and neutral particle deposition, which delivers fluorine-rich molecular fragments from PTFE ablation.

In the electrode-perpendicular plane (Figure 14a), the *F*/*C* ratio decreases with increasing deposition angle from 0° to 30°, reaches a minimum at 30°, and then increases with further increasing angle, peaking at 90°. Films on the cathode side consistently show higher *F*/*C* ratios than anode-side counterparts at equivalent angles, corroborating the cathode-biased plasma asymmetry measured by Langmuir probes and high-speed imaging.

Contrary to the expectation that the highest ion flux at 0° would cause the strongest fluorine sputtering, the *F*/*C* minimum occurs at 30° rather than at 0°. This apparent contradiction is resolved by delayed ablation: residual PTFE thermal decomposition after the main discharge generates a neutral species flux—predominantly C_2_F_4_—that peaks near the centerline and diminishes with angle (Section 3.1) [26,35]. These neutrals deliver CF_2_-rich fragments to the growing film, partially offsetting ion-induced fluorine loss. Consequently, the *F*/*C* minimum occurs at 30° rather than at the axis, reflecting the interplay between ion bombardment and neutral deposition across different angular ranges.

In the electrode-parallel plane (Figure 14b), The *F*/*C* ratio of films deposited at different angles reaches minima at ±15° and increases at larger angles. This mirrors the plasma distribution: intense ion flux is confined to within ±10–15° of the axis, where preferential fluorine sputtering is most effective; beyond this region, neutral deposition dominates, leading to higher fluorine retention. The quasi-symmetric ion flux distribution produces symmetric bond distributions, while the monotonic decrease in C–CF_x_/C–O and C–F bonds with angle reflects the gradual weakening of ion bombardment and increasing contribution of neutral species. The higher *F*/*C* in this plane is consistent with the higher CF_2_ fractions observed in Figure 12.

In summary, the angular and planar variations in *F*/*C* directly manifest the spatially varying plasma conditions quantified in Section 3.2. The competition between ion bombardment and neutral deposition, modulated by the unique plasma asymmetry in each plane, governs both the fluorine-to-carbon ratio and the underlying bond configurations. These compositional trends provide the essential link between the plasma environment and the film optical properties discussed in Section 3.7.

### 3.5. Angular Distribution of Deposited Film Thickness

**Figure 15:**
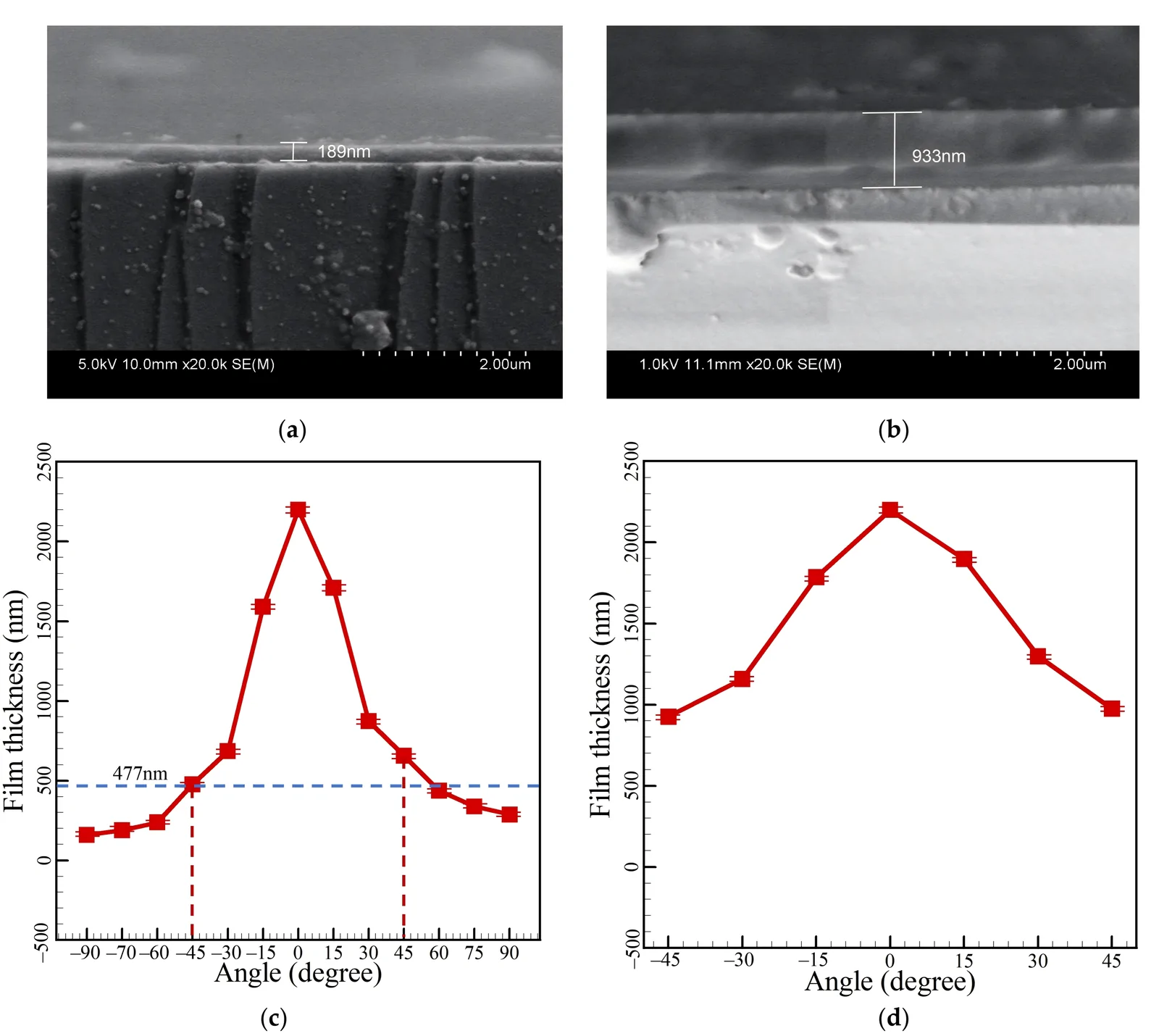
Representative cross-sectional SEM micrographs for film-thickness evaluation and angular-dependence plots of deposited-film thickness. (**a**) Electrode-perpendicular plane at 90°; (**b**) electrode-parallel plane at 45°; (**c**) angular dependence of film thickness in the electrode-perpendicular plane; (**d**) angular dependence of film thickness in the electrode-parallel plane.

Figure 15 presents representative cross-sectional SEM micrographs of films deposited at 90° in the electrode-perpendicular plane and at 45° in the electrode-parallel plane. Cross-sectional SEM shows that in the electrode-perpendicular plane, film thickness gradually decreases with increasing angle. As shown in Figure 15c, within ±45° of the thruster centerline, the film thickness exceeds 477 nm (the minimum value within this range, observed at the 45° position on the cathode side), significantly greater than at 60–90°. This suggests that the majority of the deposited mass is concentrated within a 45° divergence half-angle under the present operating conditions. At each angle, anode-side films are thicker than cathode-side films, consistent with asymmetric plasma/neutral particle distribution in this plane. Thickness values are averages of five cross-sectional measurements per angle, with standard deviations ranging from 10 to 70 nm depending on the angle.

In the electrode-parallel plane, due to the absence of electrode obstruction, film thickness decreases monotonically with angle. Consequently, films in this plane are thicker than those at corresponding angles in the perpendicular plane.

### 3.6. Surface Morphology of Deposited Films

**Figure 16:**
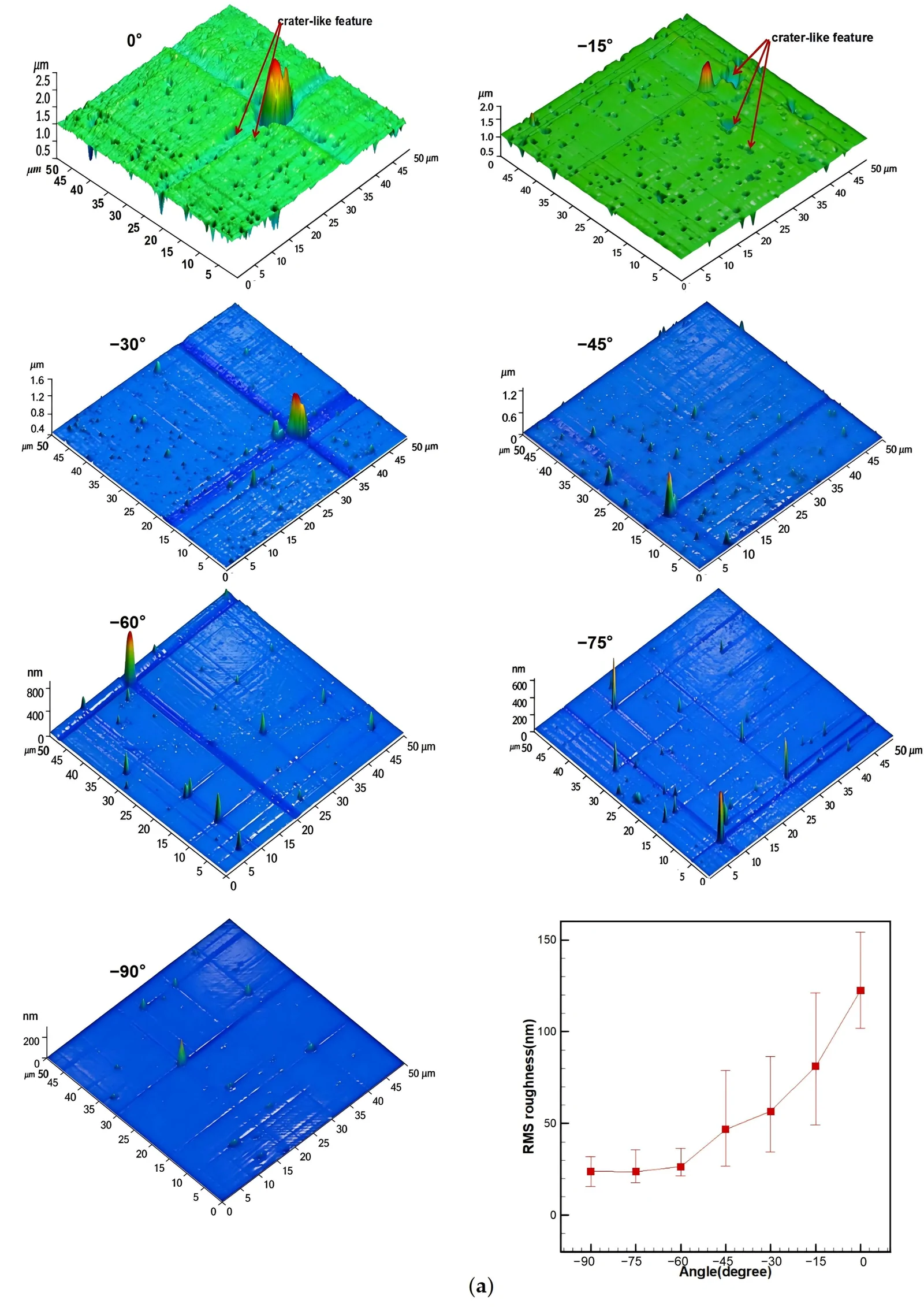

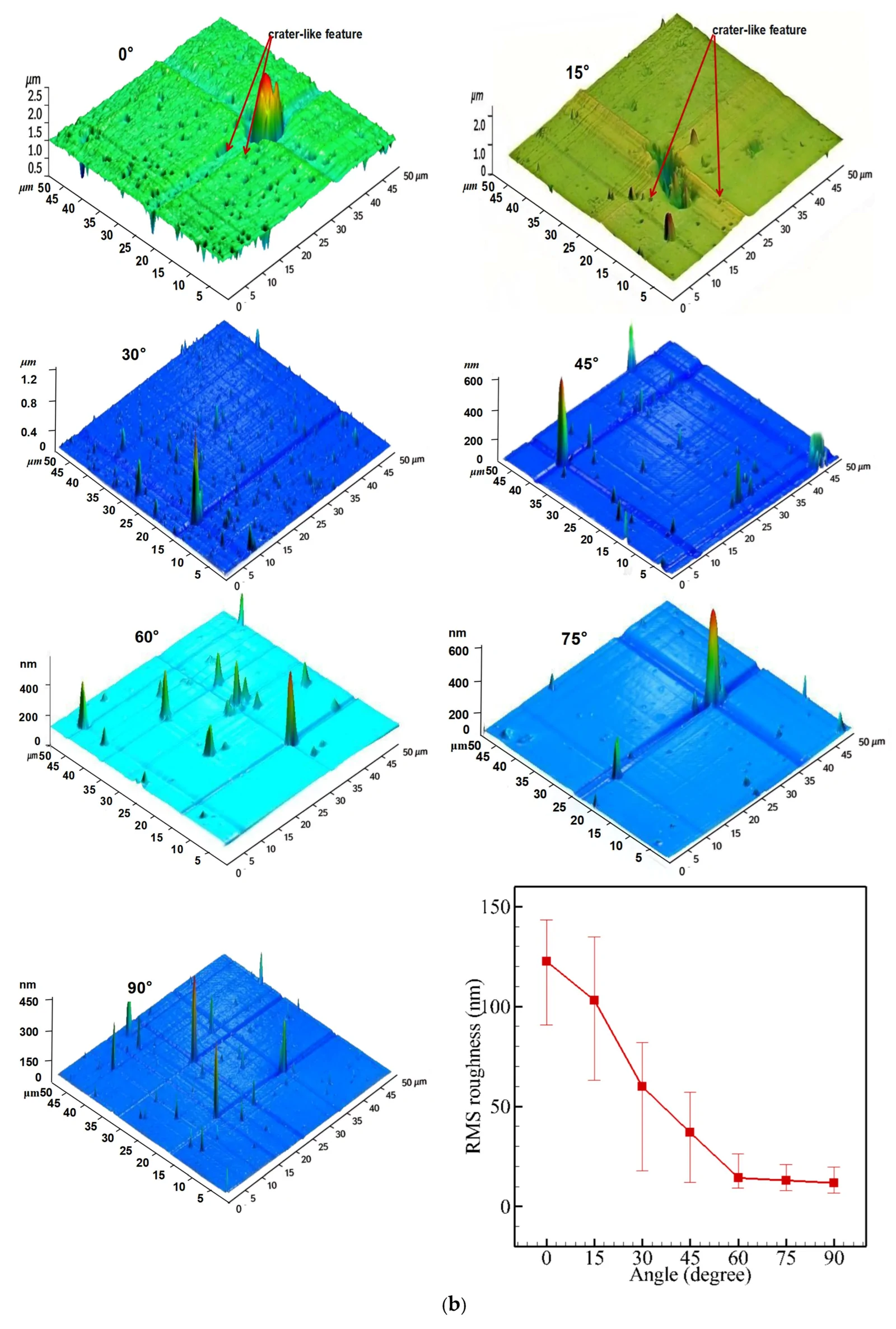

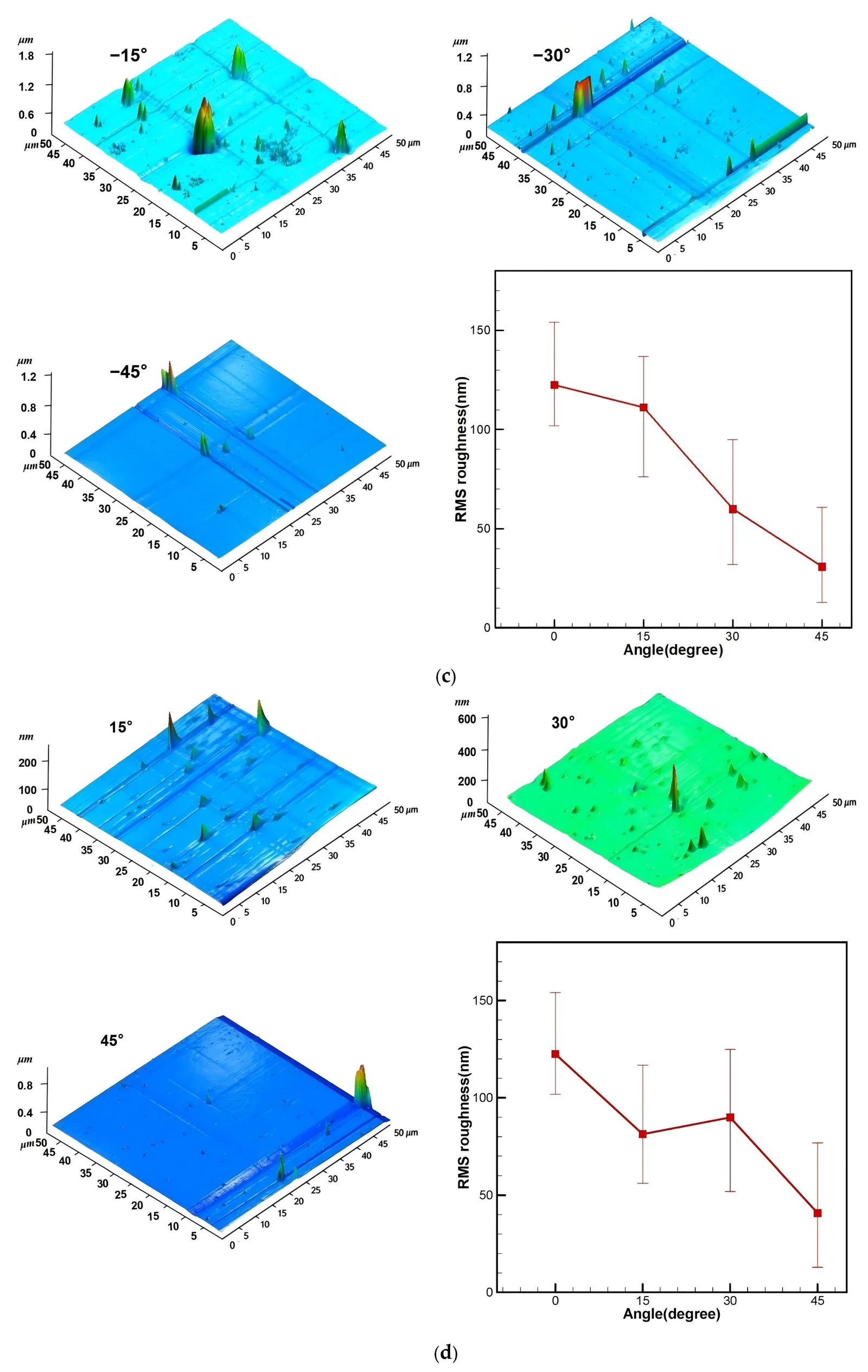
SPM images and surface roughness of fluorocarbon films as a function of deposition angle. (**a**) Cathode side (electrode-perpendicular plane). (**b**) Anode side (electrode-perpendicular plane). (**c**) Left side (electrode-parallel plane). (**d**) Right side (electrode-parallel plane).

The surface morphology reflects the interplay between deposition and etching during film growth. Figure 16 presents representative SPM images and corresponding *R*_q_ values for films at various angles in both planes.

In the electrode-perpendicular plane, films near the axis (0–15°) exhibit rough surface (*R_q_* ~120 nm at 0°) with crater-like features (shallow, bowl-shaped depressions with diameters of several micrometers), typical of energetic ion bombardment. As angle increases to 45°, the surface becomes smoother (*R_q_* ~40–50 nm), correlating with declining ion flux: weaker ion bombardment reduces etching and surface damage, allowing a more continuous film to form. Beyond 60°, surfaces show loosely packed granular deposits, likely originating from neutral particle agglomerates generated during delayed ablation [25]; *R_q_* values (~30–50 nm) remain above the bare substrate baseline (7.9 nm) due to these particulates.

In the electrode-parallel plane, surface morphology is more uniform, with fewer crater-like features. *R_q_* values (~50 to 100 nm) are generally higher than at corresponding angles in the perpendicular plane. This difference arises because the absence of electrode obstruction allows a greater flux of neutral particulates to reach the substrates, whereas intense ion flux is confined to within ±10–15° (Section 3.2). Consequently, films in this plane grow with a higher density of embedded particulates, increasing surface roughness despite weaker ion bombardment.

These morphological trends are consistent with the compositional variations (Section 3.4). In the electrode-perpendicular plane, regions with stronger ion bombardment (0–30°) exhibit both lower *F*/*C* (due to preferential fluorine sputtering [38]) and higher surface roughness (due to ion-induced etching [39]), indicating that ion flux simultaneously governs the chemical composition and morphological evolution of the films. Conversely, at larger angles (>45°), weaker ion bombardment enhances fluorine retention and reduces surface roughness, consistent with the transition to a neutral-dominated deposition regime (Section 3.2).

### 3.7. Chemo-Optical Properties of Films: Correlation with Composition and Microstructure

**Figure 17:**
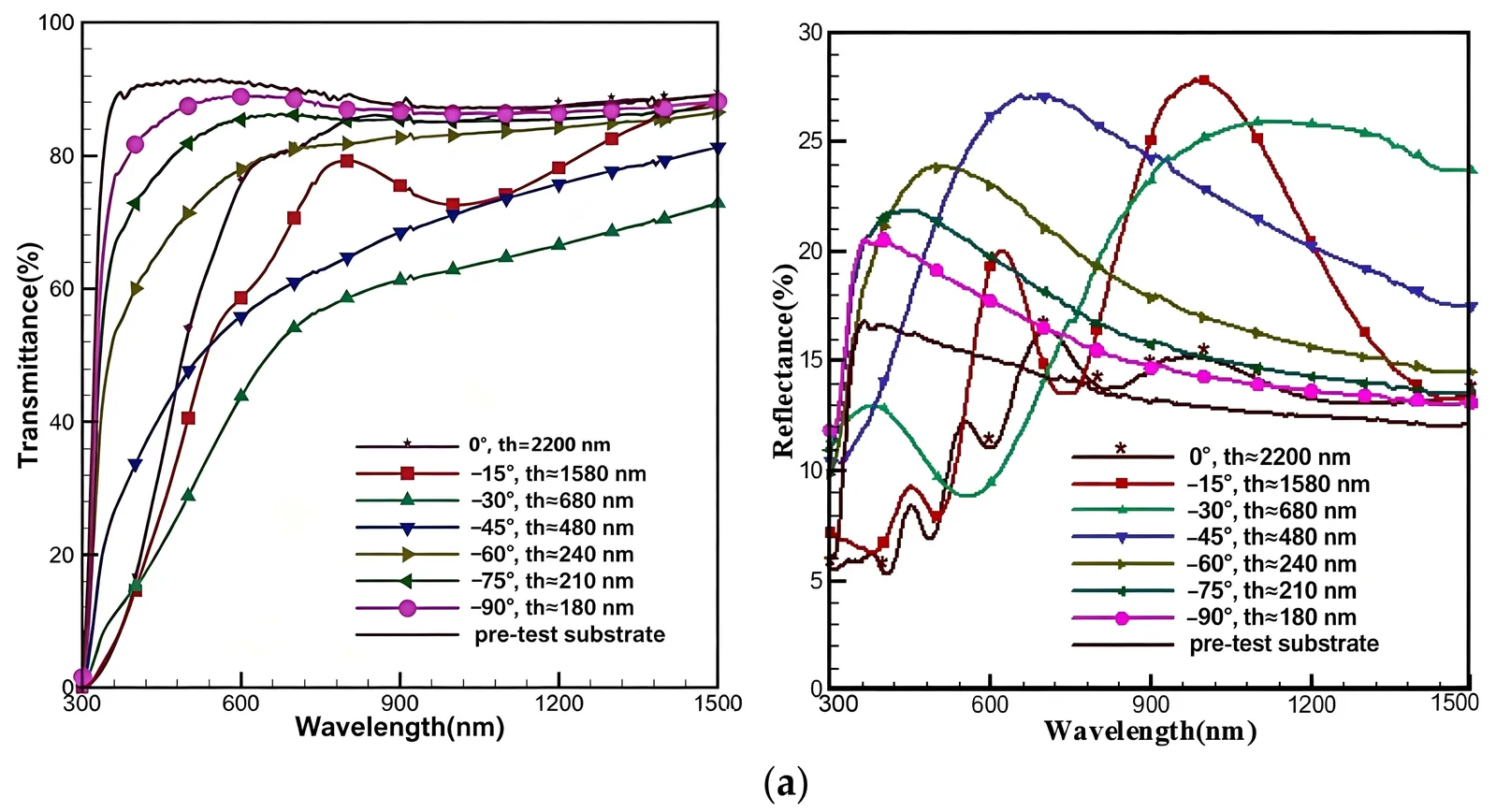

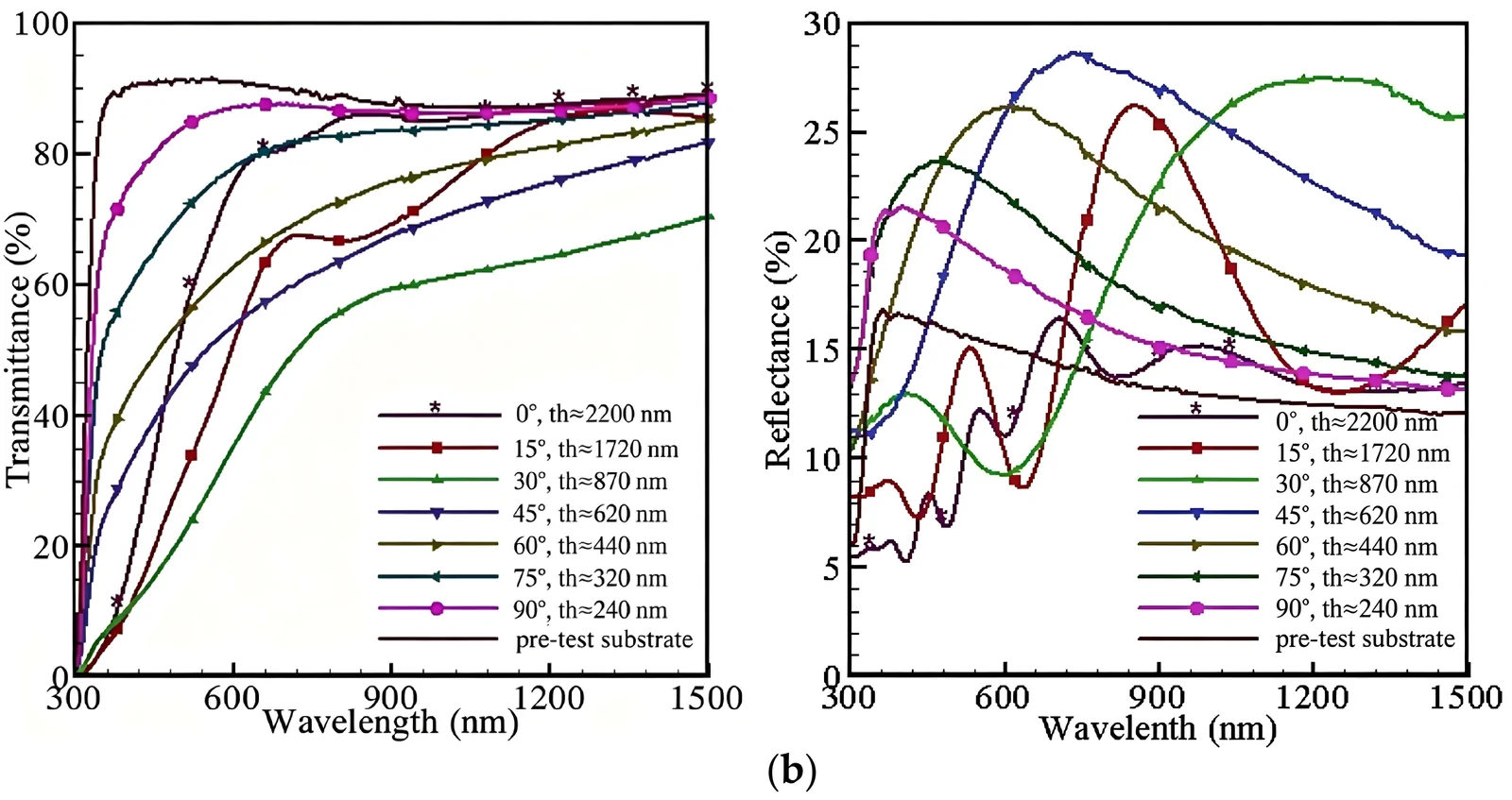
Optical properties of films in the electrode-perpendicular plane. (**a**) Cathode-side transmittance and reflectance spectra. (**b**) Anode-side transmittance and reflectance spectra.

**Figure 18:**
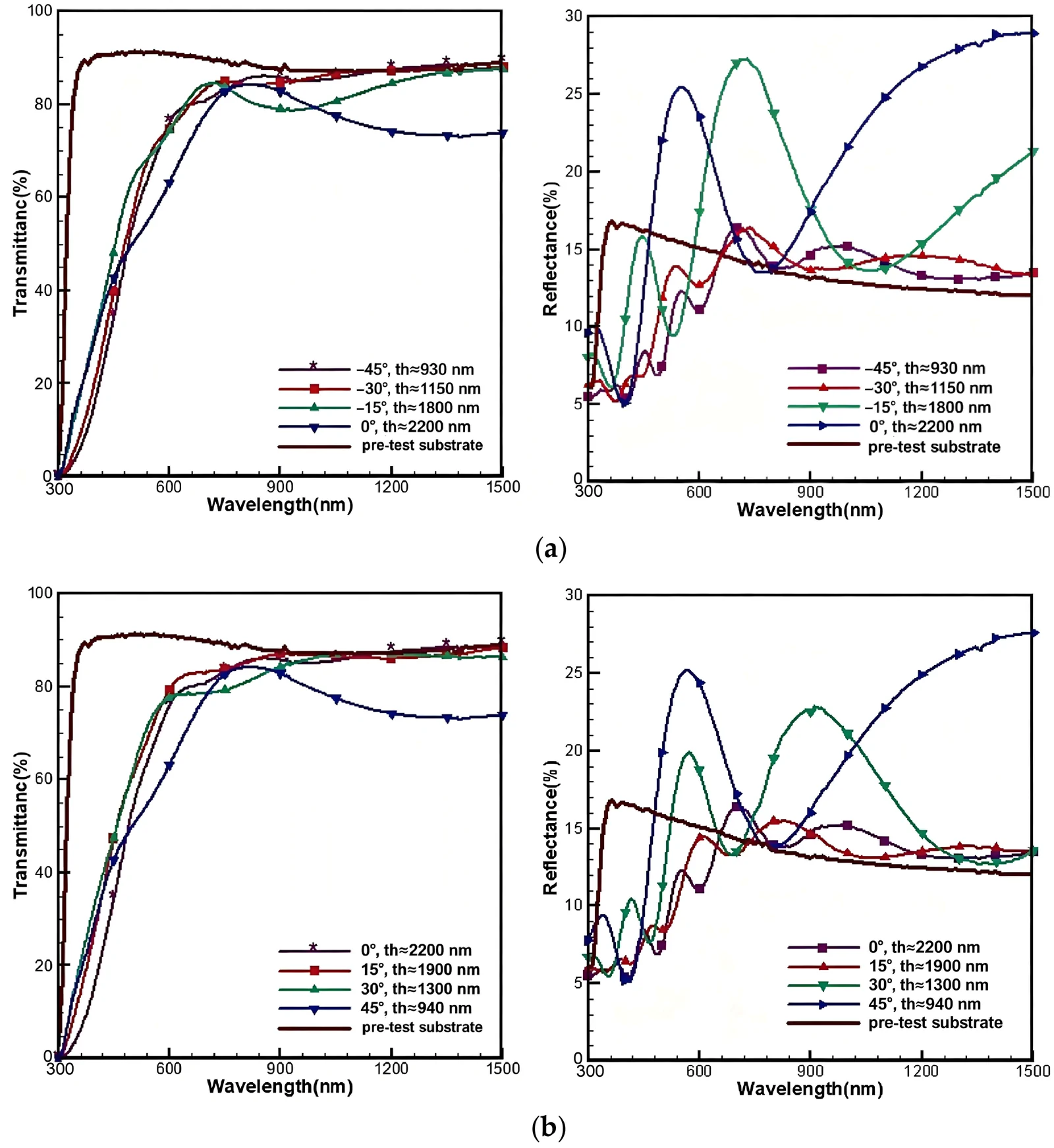
Optical properties of films in the electrode-parallel plane. (**a**) Left-side transmittance and reflectance spectra. (**b**) Right-side transmittance and reflectance spectra.

Figure 17 and Figure 18 present the transmittance and reflectance spectra (300–1500 nm) of fluorocarbon films deposited at various angular positions in the two orthogonal planes, together with reference spectra of bare glass substrates. Both transmittance and reflectance exhibit systematic angular dependence, with distinct characteristics between the electrode-perpendicular and electrode-parallel planes. To facilitate direct correlation between optical performance and film geometry, each spectrum in the figure legends is annotated with the corresponding film thicknessdetermined from cross-sectional SEM.

The optical response of these films arises from three coupled factors: intrinsic absorption governed by chemical bonding, geometrical thickness-dependent optical path length, and surface-morphology-induced scattering. All three are directly governed by the spatially varying plasma conditions—specifically, the competing fluxes of energetic ions and neutral ablation fragments, which are known to simultaneously modify surface morphology and fluorine content [38,39]—as established in Section 3.2, Section 3.3, Section 3.4, Section 3.5 and Section 3.6. This multi-parameter interplay produces the anisotropic chemo-optical responses observed across the plume angular range.

In the electrode-perpendicular plane (Figure 17), a distinct positive correlation between the *F*/*C* ratio and optical transmittance is established. At identical angular offsets, cathode-side films exhibit higher *F*/*C* ratios and consistently higher transmittance than their anode-side counterparts. This correlation can be understood from established fluorocarbon film physics: first, higher fluorine content intrinsically reduces the effective refractive index of fluorocarbon films, thereby decreasing the index contrast at the film–glass interface and suppressing Fresnel reflection [40,41]. Second, anode-side films—with their lower *F*/*C* ratios—exhibit a pronounced red-shift of the UV-Vis absorption edge and systematically lower transmittance, particularly in the shorter-wavelength region. This spectral evolution is widely associated with carbon-enriched domains and increased sp^2^-hybridized character, which are known to narrow the optical bandgap [41,42,,43,44]. Across the entire 0–90° angular range, elevated fluorine content blue-shifts the UV absorption edge and improves transparency, while reduced *F*/*C* shifts the absorption edge toward longer wavelengths and degrades transmittance.

A critical experimental decoupling effect between geometrical thickness and optical loss is observed at 30° on the anode side. If only thickness were considered, the lowest transmittance would be expected at 0°, where the film thickness reaches its maximum (~2200 nm). However, the minimum transmittance occurs at 30°, where the film thickness is merely ~870 nm (Section 3.5) but the *F*/*C* ratio reaches its minimum and the C–C/C–H bond fraction reaches its maximum (Section 3.4). The pronounced red-shift of the absorption edge at this angle strongly indicates enhanced intrinsic absorption driven by compositional changes. This comparison provides direct evidence that, within the investigated angular range, chemical-composition-driven optical absorption can dominate over the geometrical thickness effect.

Reflectance spectra provide complementary information on interfacial and morphological characteristics. In the 0–30° angular sector, persistent intense ion bombardment generates high surface roughness with abundant ion-etched craters on both cathode and anode sides. These irregular topographies distort thin-film interference fringes and introduce complex oscillatory features in reflectance spectra (Figure 17b), accompanied by diffuse scattering losses. At 45–90°, attenuated deposition flux leads to reduced film thickness and suppressed surface roughness, yielding more regular reflectance profiles. For reflectance, roughness exerts a more direct influence: surface irregularities perturb the coherent interference condition, producing pronounced spectral oscillations that are not observed in smoother films. This distinction—transmittance responding more directly to compositional variations, while reflectance is more sensitive to surface morphology—is consistent with the behavior reported for fluorocarbon films [41,45].

In the electrode-parallel plane (Figure 18), the transmittance spectra on the left and right sides of the thruster are nearly identical, exhibiting a quasi-symmetric distribution. This reflects the symmetric deposition flux distribution in this plane (Section 3.2). The overall transmittance values are higher than those at corresponding angles in the electrode-perpendicular plane, which is attributed to the relatively higher *F*/*C* ratios of parallel-plane films (Section 3.4). However, due to the relatively higher surface roughness in this plane, the reflectance spectra exhibit complex features. This observation further corroborates that reflectance is considerably more sensitive to surface morphological perturbations than transmittance.

In summary, the present results demonstrate that the optical degradation of APPT contaminant films is governed by the coupled contributions of chemical bonding, film thickness, and surface morphology, with their relative weights varying with deposition angle and the specific optical channel (transmittance vs. reflectance) considered. From an engineering perspective, the established chemo-optical correlation provides practical guidance for risk-based layout optimization of pulsed plasma thrusters and adjacent optical-sensitive payloads.

## 4. Conclusions

By integrating spatially resolved plasma diagnostics with multi-technique materials characterization, this study establishes direct quantitative links between anisotropic plasma conditions and the chemo-optical properties of fluorocarbon films deposited from an APPT plume. The main findings are:

1. Plasma anisotropy defines the deposition landscape. The APPT discharge evolves through four phases: current sheet formation with Hall-effect-induced canting, magnetic pinch with asymmetric plasma development, reverse-charging re-striking, and neutral-dominated expansion (delayed ablation). In the electrode-perpendicular plane, electron density *n_e_* is cathode-biased (peak ~8 × 10^20^ m^−3^ at 0–10°) and electron temperature *T_e_* is anode-biased (peak ~5 eV). The electrode-parallel plane exhibits axial symmetry, with high *n_e_* confined within ±10°. These anisotropic plasma conditions create spatially non-uniform deposition environments that govern all subsequent film properties.
2. Ion–neutral competition governs chemical composition and bonding. The *F*/*C* ratio ranges from 0.6 to 1.0, far below that of pristine PTFE (2.0). In the electrode-perpendicular plane, the *F*/*C* ratio decreases with increasing deposition angle from 0° to 30°, reaches a minimum of ~0.6 at 30°, and then increases as the angle increases further to 90°. In the electrode-parallel plane, *F*/*C* minima occur at ±15°. Cathode-side films consistently show higher *F*/*C* than anode-side films, directly corroborating the cathode-biased *n_e_*. Contrary to the expectation that the highest ion flux at 0° would minimize fluorine retention, the *F*/*C* minimum occurs at 30° rather than at 0°. This is explained by delayed ablation: neutral C_2_F_4_ preferentially deposits near the axis, offsetting fluorine sputtering. This ion–neutral competition also governs bond configurations, as reflected in the opposite variations in C–C/C–H and CF_2_ fractions.
3. Chemo-optical anisotropy arises from coupled contributions of chemical bonding, film thickness, and surface morphology. Transmittance exhibits a strong positive correlation with *F*/*C* ratio. The minimum transmittance occurs at 30° in the electrode-perpendicular plane, where *F*/*C* reaches its lowest value and the C–C/C–H bond fraction reaches its maximum. Notably, this transmittance minimum does not occur at 0°, despite 0° having the maximum film thickness. This observation indicates that, within the investigated angular range, chemical-composition-driven optical absorption can be sufficiently large to dominate over the geometrical thickness effect. For the angle-dependent optical behavior observed in this work, surface roughness distorts specular reflectance by perturbing thin-film interference, whereas angular variations in transmittance respond more directly to compositional effects. In the electrode-parallel plane, higher *F*/*C* ratios yield generally higher transmittance, while the elevated surface roughness strongly disturbs reflectance spectra. The multi-parameter correlation thus indicates that all three factors—bond configuration, film thickness, and surface roughness—co-determine the optical response.
4. Design guideline for microsatellites. To avoid transmittance dropping below 20% at 550 nm or significant reflectance alteration, optical surfaces within a 30° half-angle in the electrode-perpendicular plane require active shielding. In the electrode-parallel plane, a ±15° half-angle requires similar protection. These quantitative angular boundaries, grounded in the chemo-optical mechanisms established above, provide a risk-based criterion for the layout optimization of thrusters and adjacent optical payloads.

In summary, this work demonstrates that spatially varying plasma conditions within an APPT plume, acting through the interplay between ion bombardment and neutral-species deposition, dictate the anisotropic chemo-optical properties of PTFE-derived fluorocarbon films. Using plasma diagnostics, we reveal how these plasma conditions govern film-formation behavior, which in turn shapes the films’ chemical structure and morphology and ultimately drives their chemo-optical anisotropy. The established plasma–structure–property framework enables quantitative contamination risk assessment for microsatellite components and lays a foundation for optimizing thruster design and developing mitigation strategies, and offers a transferable framework for other plasma deposition processes (e.g., PECVD and DLC) and methodological relevance to laser-based transfer processes (e.g., LIFT and LIBT). Future work will extend this framework to broader operating parameters and employ spectroscopic ellipsometry under controlled deposition conditions to accurately determine the optical constants of APPT contaminant films, enabling quantitative separation of the individual optical loss channels for more reliable contamination simulations.

## Data Availability

Data is contained within the article.
